# A pathway for chitin oxidation in marine bacteria

**DOI:** 10.1038/s41467-022-33566-5

**Published:** 2022-10-06

**Authors:** Wen-Xin Jiang, Ping-Yi Li, Xiu-Lan Chen, Yi-Shuo Zhang, Jing-Ping Wang, Yan-Jun Wang, Qi Sheng, Zhong-Zhi Sun, Qi-Long Qin, Xue-Bing Ren, Peng Wang, Xiao-Yan Song, Yin Chen, Yu-Zhong Zhang

**Affiliations:** 1 State Key Laboratory of Microbial Technology, Shandong University, Qingdao, China; 2 College of Marine Life Sciences, Ocean University of China, Qingdao, China; 3 Laboratory for Marine Biology and Biotechnology, Pilot National Laboratory for Marine Science and Technology, Qingdao, China; 4 School of Life Sciences, University of Warwick, Coventry, CV4 7AL United Kingdom; 5 Marine Biotechnology Research Center, State Key Laboratory of Microbial Technology, Shandong University, Qingdao, China

**Keywords:** Environmental microbiology, Bacterial physiology, Polysaccharides, Marine microbiology

## Abstract

Oxidative degradation of chitin, initiated by lytic polysaccharide monooxygenases (LPMOs), contributes to microbial bioconversion of crystalline chitin, the second most abundant biopolymer in nature. However, our knowledge of oxidative chitin utilization pathways, beyond LPMOs, is very limited. Here, we describe a complete pathway for oxidative chitin degradation and its regulation in a marine bacterium, *Pseudoalteromonas prydzensis*. The pathway starts with LPMO-mediated extracellular breakdown of chitin into C1-oxidized chitooligosaccharides, which carry a terminal 2-(acetylamino)−2-deoxy-D-gluconic acid (GlcNAc1A). Transmembrane transport of oxidized chitooligosaccharides is followed by their hydrolysis in the periplasm, releasing GlcNAc1A, which is catabolized in the cytoplasm. This pathway differs from the known hydrolytic chitin utilization pathway in enzymes, transporters and regulators. In particular, GlcNAc1A is converted to 2-keto-3-deoxygluconate 6-phosphate, acetate and NH_3_ via a series of reactions resembling the degradation of D-amino acids rather than other monosaccharides. Furthermore, genomic and metagenomic analyses suggest that the chitin oxidative utilization pathway may be prevalent in marine Gammaproteobacteria.

## Abstract (web summary)

Lytic polysaccharide monooxygenases contribute to microbial degradation of chitin, but how the resulting oxidized chitooligosaccharides are utilized by microbes is unclear. Here, the authors describe a complete pathway for oxidative chitin utilization in marine bacteria.

## Introduction

Chitin, an insoluble linear polysaccharide of β-1,4 linked *N*-acetyl-D-glucosamine (GlcNAc), is the second most abundant natural biopolymer after cellulose^1^. Degradation and recycling of chitin driven by marine bacteria is crucial for biogeochemical cycles of carbon and nitrogen in the oceans^2–4^. Although the global production of chitin is estimated to be approximately 10^11^ tons annually, which steadily sink to the ocean floor as ‘marine snow’, no substantial accumulation of chitin occurs in ocean sediments^5–7^ due to efficient degradation by marine heterotrophic bacteria. Chitin can be degraded by bacteria through two pathways, one of which, the hydrolytic chitin utilization pathway initiated by chitinases, is well established. This hydrolytic chitin utilization pathway involves extracellular degradation of polymeric chitin into soluble chitooligosaccharides, transport of chitooligosaccharides across membranes and intracellular GlcNAc catabolism to fructose-6-P, acetate and NH_3_. This pathway is found to be largely conserved in important chitin-degrading bacteria, e.g. *Vibrionaceae*^8–10^ and others^11,12^. In contrast, the oxidative chitin utilization pathway initiated by lytic polysaccharide monooxygenases (LPMOs) remains poorly studied.

LPMOs are copper-dependent enzymes that oxidize the surfaces of crystalline polysaccharides to generate “nicks”, allowing canonical hydrolytic enzymes to depolymerize complex biomass more efficiently^13,14^. Current research on this latter pathway mainly focuses on the functional identification of LPMOs and clarification of their underlying catalytic mechanisms^15–18^. In the CAZY database^19^, chitin-active LPMOs are grouped into three auxiliary activity families AA10, AA11 and AA15, with the majority of the characterized sequences belonging to the AA10 family. A hallmark for the latter pathway is that all characterized chitin-active LPMOs can only oxidize the C1 carbons of the glycosidic bonds in chitin and produce chitooligosaccharides with a terminal oxidized sugar, 2-(acetylamino)−2-deoxy-D-gluconic acid (GlcNAc1A)^15–17,20,21^. To date, it remains unknown how oxidized chitooligosaccharides are subsequently degraded by chitin-utilizers. It is postulated that, oxidized chitooligosaccharides would be eventually converted to monosaccharides GlcNAc and GlcNAc1A by as yet unidentified glycoside hydrolases (GHs), a way akin to oxidative degradation of cellulose^22^. GlcNAc1A is a unique oxidized product from the LPMO-dependent pathway, which is more similar to *N*-acetyl-D-amino acids than GlcNAc in terms of chemical structure and charge. As such, it is tempting to speculate that bacteria may catabolize GlcNAc1A via a different pathway from that for GlcNAc.

Bacteria of the *Pseudoalteromonas* genus are exclusively of marine origin, which are widely distributed in the global marine environments from surface water to deep-sea sediments^23–25^. Some *Pseudoalteromonas* strains have been reported to be chitinolytic owing to the presence of a three-gene chitin degradation cluster (*cdc*) in their genomes, encoding two GH18 chitinases (ChiA and ChiC) and one AA10 LPMO^26,27^. Although not biochemically characterized yet, LPMOs are found in all chitinolytic *Pseudoalteromonas* strains described so far^26–29^. Therefore, we hypothesize that there is likely a pathway to metabolize oxidized LPMO products from chitin in *Pseudoalteromonas* strains. Indeed, in this study, several chitinolytic *Pseudoalteromonas* strains were found to be able to utilize GlcNAc1A, the hallmark intermediate of the oxidative chitin utilization pathway.

**Fig. 1:**
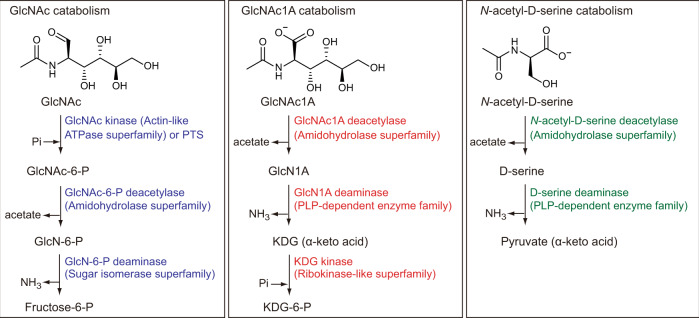
The proposed catabolic pathway for GlcNAc1A in this study and reported catabolic pathways for GlcNAc and *N*-acetyl-D-serine. Catabolism of GlcNAc is initiated by phosphorylation by a GlcNAc kinase (e.g. in *Shewanella oneidensis*^11^) or a PTS (e.g. in *Vibrionaceae*^8,40^), which is subsequently deacetylated and deaminated to produce fructose-6-P. Catabolism of *N*-acetyl-D-serine in some bacteria starts with deacetylation followed by deamination and formation of pyruvate^44,54^. In the proposed catabolic pathway for GlcNAc1A in this study, GlcNAc1A is deacetylated and deaminated directly to produce KDG without activation by phosphorylation. As such, this GlcNAc1A degradation pathway resembles *N*-acetyl-D-amino acid catabolism through deacetylation and deamination. Enzymes involved in catabolic pathways for GlcNAc1A, GlcNAc and *N*-acetyl-D-serine are shown in red, blue and green, respectively. The protein families of enzymes involved in each pathway are indicated in parentheses. PTS phosphotransferase, GlcN D-glucosamine, PLP pyridoxal 5-phosphate, KDG 2-keto-3-deoxygluconate.

Here, we show the complete pathway of oxidative chitin degradation adopted by the marine bacterium *Pseudoalteromonas prydzensis* ACAM 620, in which GlcNAc1A is catabolized via a way akin to *N*-acetyl-D-amino acid utilization (Fig. 1). This oxidative chitin utilization pathway is prevalent in marine Gammaproteobacteria based on genomic and metagenomic analyses.

## Results and discussion

### Chitinolytic *Pseudoalteromonas prydzensis* ACAM 620 could utilize GlcNAc1A

**Fig. 2:**
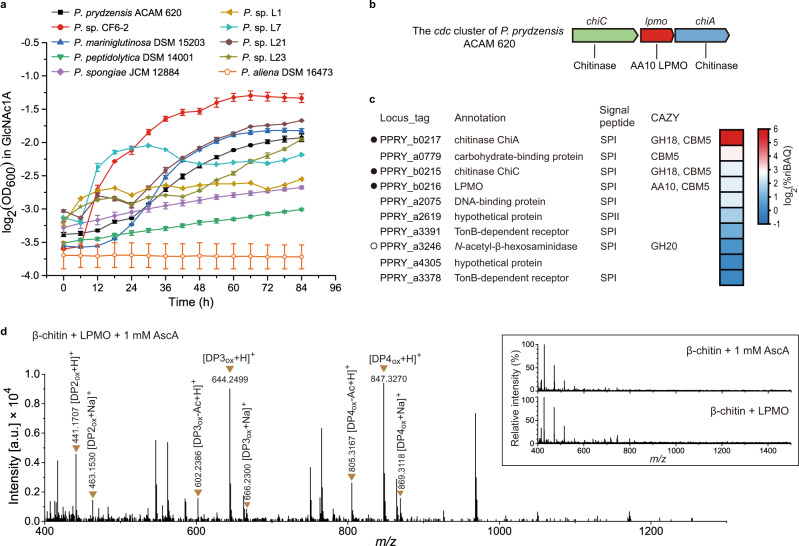
Growth of chitinolytic marine *Pseudoalteromonas* strains on GlcNAc1A and functional analysis of the recombinant AA10 LPMO from *P. prydzensis* ACAM 620. **a** Growth of chitinolytic *Pseudoalteromonas* spp. in the minimal medium supplemented with 0.2% (w/v) GlcNAc1A. The *y*-axis represents log_2_ transformation of the OD_600_ value. Data are presented as mean ± standard deviations (SD) (*n* = 2 independent experiments). **b** Genetic organization of the *cdc* cluster of *P. prydzensis* ACAM 620. **c** Heat map for the top ten most abundant proteins in the secretome of strain ACAM 620 grown on 0.5% (w/v) colloidal chitin as the sole carbon source. The colors in the heat map indicate relative protein abundance, ranging from high (red) to low abundance (blue). The data are presented as log_2_ transformation of the mean values of two biological replicates for each protein. These ten proteins account for 83.86% of the total protein abundance. The locus tag, protein annotation, type of signal peptides and CAZy family (glycosyl hydrolase (GH), carbohydrate-binding module (CBM) and auxiliary activity (AA)) are shown. Chitinolytic enzymes encoded by the *cdc* cluster are marked by solid circles and the other one by an empty circle. SpI, signal peptidase I cleavage site; SpII, signal peptidase II cleavage site. **d** Positive-mode Q-TOF-MS spectrum of products generated by the AA10 LPMO from strain ACAM 620 acting on 0.2% (w/v) squid pen β-chitin in the presence of 1 mM AscA. The insets show the negative control reactions without either LPMO or AscA, which did not generate detectable amounts of oxidized chitooligosaccharides. 100% relative intensity in the inserts represents 9.1 ×10^3^ (control reaction without LPMO) and 7.0 ×10^3^ (control reaction without AscA) arbitrary units (a.u.), respectively. Theoretical masses of relevant products are listed in Supplementary Table 2. DP, degree of polymerization; subscript OX, C1-oxidized chitooligosaccharides with a GlcNAc1A moiety; DP3_ox_-Ac, DP3_ox_ lacking one acetyl group; DP4_ox_-Ac, DP4_ox_ lacking one acetyl group. The graph shows a representative MS spectrum of at least three independent replicates. Source data are provided as a Source Data file.

Metagenomic and genomic analyses suggest that *Pseudoalteromonas* is an important marine bacterial group containing AA10 LPMOs that are known to initiate the first step of oxidative chitin degradation (Supplementary Fig. 1). To investigate this as yet uncharacterized pathway in *Pseudoalteromonas*, we selected 19 chitinolytic strains from over 13 species that all contain at least one AA10 LPMO-encoding gene localized in the *cdc* cluster (Supplementary Table 1). We examined the growth of these strains on GlcNAc1A, a unique product from chitin degradation through the oxidative pathway, as the sole carbon source. The growth of these strains on GlcNAc and D-gluconate was also examined which was used as the control. GlcNAc is a product from chitin degradation through the hydrolytic pathway, whereas D-gluconate (a structural analogue of GlcNAc1A) is a key intermediate in the Entner-Doudoroff pathway of some bacteria^30^. While all strains grew well on GlcNAc (Supplementary Table 1), only nine strains had noticeable growth on GlcNAc1A (Fig. 2a and Supplementary Table 1), suggesting that a complete oxidative chitin utilization pathway is likely present in these GlcNAc1A-utilizing strains. None of these chitinolytic strains could utilize D-gluconate as the sole carbon source (Supplementary Table 1), suggesting that GlcNAc1A is unlikely converted to D-gluconate in these GlcNAc1A-utilizing strains. To reveal the oxidative chitin utilization pathway in chitinolytic *Pseudoalteromonas* strains, *P. prydzensis* ACAM 620, isolated from Antarctic sea ice but capable of growing optimally at 22–25 °C^31^, was selected for further characterization since it can grow on GlcNAc1A efficiently (Fig. 2a).

### Strain ACAM 620 secrets an AA10 LPMO to cleave chitin into C1-oxidized chitooligosaccharides

Strain ACAM 620 harbors only one AA10 LPMO from the *cdc* cluster (Fig. 2b), which is phylogenetically distantly related to characterized terrestrial AA10 homologs (Supplementary Fig. 2). When grown on chitin, strain ACAM 620 secreted a considerable amount of the LPMO protein in the culture medium (Fig. 2c, Supplementary Fig. 3 and Supplementary Data 1), suggesting that LPMO is likely involved in the extracellular chitin degradation. To further ascertain the function of LPMO in chitin degradation, the *lpmo* gene from strain ACAM 620 was overexpressed in *Escherichia coli* BL21 (DE3) and recombinant LPMO was extracted from its periplasmic fraction by osmotic shock and purified (Supplementary Fig. 4a). In the presence of an external electron donor such as ascorbic acid (AscA), the recombinant LPMO could degrade crystalline α- and β-chitin using O_2_ or H_2_O_2_ as the co-substrate but showed no detectable activity towards cellulose (Fig. 2d and Supplementary Fig. 4b–e), indicating its chitin specificity. High-Resolution Q-TOF mass spectrometry (Q-TOF-MS) analysis revealed that it oxidized the C1 carbons of chitin to produce chitooligosaccharides with a terminal GlcNAc1A moiety (Fig. 2d, Supplementary Fig. 4b and c and Supplementary Table 2), similar to reported chitin-active LPMOs^13,15,21^. The catalytic activity of this LPMO depends on extracellular electron donors (Fig. 2d and Supplementary Fig. 4b and c). However, unlike fungal cellulose-active LPMOs^32,33^, no enzymatic redox partner has been found for bacterial LPMOs yet, and the natural electron donor for bacterial LPMOs is still unclear so far. In addition to LPMO, strain ACAM 620 also secreted functional chitinases such as ChiA and ChiC when grown on chitin (Fig. 2c and Supplementary Figs. 3 and 5). Thus, a synergistic action between LPMO and chitinases likely occurred in the extracellular degradation of chitin in strain ACAM 620, leading to the efficient breakdown of chitin.

### Strain ACAM 620 contains an eight-gene cluster essential for utilizing oxidized chitooligosaccharides

**Fig. 3:**
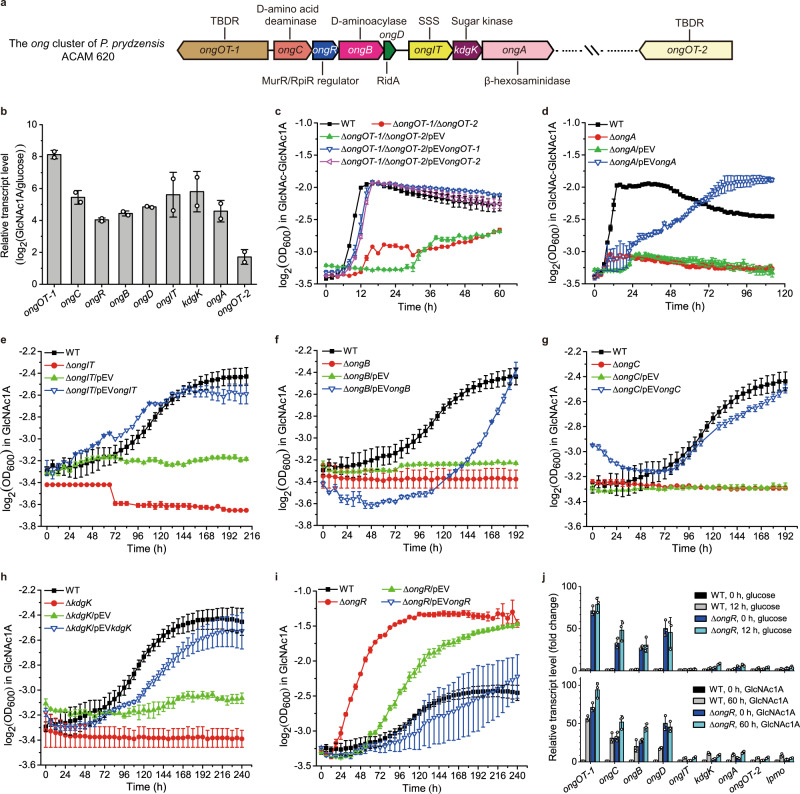
Identification of key genes in strain ACAM 620 involved in utilizing oxidized chitooligosaccharides based on transcriptomic and genetic analyses. **a** Genetic organization of the *ong* cluster of strain ACAM 620. TBDR, TonB-dependent receptor; SSS, sodium solute symporter. **b** RNA-seq assay of the transcriptions of genes from the *ong* cluster and *ongOT-2* in strain ACAM 620 in the minimal medium supplemented with 0.2% (w/v) GlcNAc1A. Values are expressed as fold change (log_2_) compared to cultures in the minimal medium supplemented with 0.2% (w/v) glucose. Data are presented as mean ± SD (*n* = 2 independent experiments). **c–i** The growth phenotype of strain ACAM 620 with a single gene deletion in the *ong* cluster on GlcNAc1A or GlcNAc-GlcNAc1A as the sole carbon source. Wild-type strain (WT), mutant strains and complemented strains of mutants were grown at 25 °C in the minimal medium supplemented with 10 mM GlcNAc1A or 10 mM GlcNAc-GlcNAc1A. Deletion mutant strains with the empty plasmid pEV were used a control. The *y*-axes in **c-i** represent log_2_ transformation of the OD_600_ value. Data are presented as mean ± SD (*n* = 2 independent experiments). **j**, RT-qPCR assay of the transcriptions of *lpmo*, *ongOT-2* and genes from the *ong* cluster in the WT and △*ongR* mutant strains in response to 0.2% (w/v) glucose (upper) or 0.2% (w/v) GlcNAc1A (lower) in the minimal medium. Values are expressed as fold change compared to precultures of the WT strain in the minimal medium supplemented with 0.2% (w/v) glucose. The *rpoD* gene was used as an internal reference. Data are presented as mean ± SD (*n* = 3 independent experiments). Source data are provided as a Source Data file.

To uncover genes responsible for utilizing oxidized chitooligosaccharides in strain ACAM 620, we performed RNA-seq experiments on strain ACAM 620 grown on GlcNAc1A and glucose as the sole carbon source, respectively. Transcriptomic analysis showed that the transcripts of eight genes that compose an uncharacterized gene cluster were all significantly upregulated in strain ACAM 620 grown on GlcNAc1A, but not on glucose (Fig. 3a and b and Supplementary Table 3). Expression of the genes from this gene cluster was also induced by chitin (Supplementary Fig. 6). This gene cluster was annotated to encode two transporters, one MurR/RpiR family transcriptional regulator, one β-hexosaminidase, one sugar kinase, one RidA family protein, one putative D-aminoacylase and one putative D-amino acid deaminase (Supplementary Table 3). This gene cluster is present in the genomes of all GlcNAc1A-utilizing *Pseudoalteromonas* strains, but absent from 8 of 10 strains incapable of utilizing GlcNAc1A (Supplementary Table 1), suggesting that this cluster is possibly involved in the oxidative degradation of chitin. Further genetic analyses of strain ACAM 620 in vivo showed that, except for the Δ*ongOT-1* and Δ*ongR* mutant strains, none of the other mutant strains with a single gene deletion from the identified cluster were able to grow on GlcNAc1A or GlcNAc-GlcNAc1A, but their growth was almost fully restored to the wild-type levels by complementation of corresponding genes (Fig. 3 and Supplementary Figs. 7 and 8a), confirming the key role of this gene cluster in catabolizing oxidized chitooligosaccharides. We term this gene cluster as the *ong* cluster in this study.

### Transport of oxidized chitooligosaccharides across outer membrane

TonB-dependent receptors (TBDRs) are outer membrane transporters, which have been reported to be involved in the uptake of phytoplankton-derived polysaccharides such as alginate, xylan and laminarin^34^. The *ong* cluster in strain ACAM 620 encodes a potential outer-membrane TBDR, OngOT-1, sharing no significant sequence identity to reported TBDRs. In addition to OngOT-1, transcriptomic analysis revealed that the transcript of *ongOT-2*, an *ongOT-1* paralog localized elsewhere in the genome, was also significantly upregulated in strain ACAM 620 grown on GlcNAc1A (Fig. 3a and b). OngOT-2 had 32% sequence identity to OngOT-1. Both proteins were detected in the secretome of strain ACAM 620 grown on chitin (Supplementary Table 3 and Supplementary Data 1). The *ongOT-1* and *ongOT-2* genes were then deleted from strain ACAM 620 to construct a double-deletion mutant (Δ*ongOT-1*/Δ*ongOT-2*). The growth of Δ*ongOT-1*/Δ*ongOT-2* mutant strain on GlcNAc-GlcNAc1A was severely impaired, but fully restored by complementation of either of the two genes (Fig. 3c), indicating that both OngOT-1 and OngOT-2 can import oxidized chitooligosaccharides in strain ACAM 620. Moreover, the Δ*ongOT-1*/Δ*ongOT-2* mutant strain showed no difference in growth from the wild-type strain on (GlcNAc)_2_, (GlcN)_2_, GlcNAc, GlcN (D-glucosamine) or *N*-acetylmuramic acid (MurNAc) (Supplementary Fig. 8), suggesting that OngOT-1 and OngOT-2 are not involved in the uptake of these carbohydrates. These data indicate that OngOT-1 and OngOT-2 specifically transport oxidized chitooligosaccharides across outer membrane to the periplasm of strain ACAM 620. This is somewhat different to the transport of chitooligosaccharides in bacteria resulting from the hydrolytic chitin utilization pathway, whereby chitooligosaccharides are transported into the periplasm via a specific porin in *Vibrionaceae*^35^ and a predicted TBDR in *Shewanella oneidensis*^11^.

### Hydrolysis of oxidized chitooligosaccharides into GlcNAc1A and GlcNAc in the periplasm

**Fig. 4:**
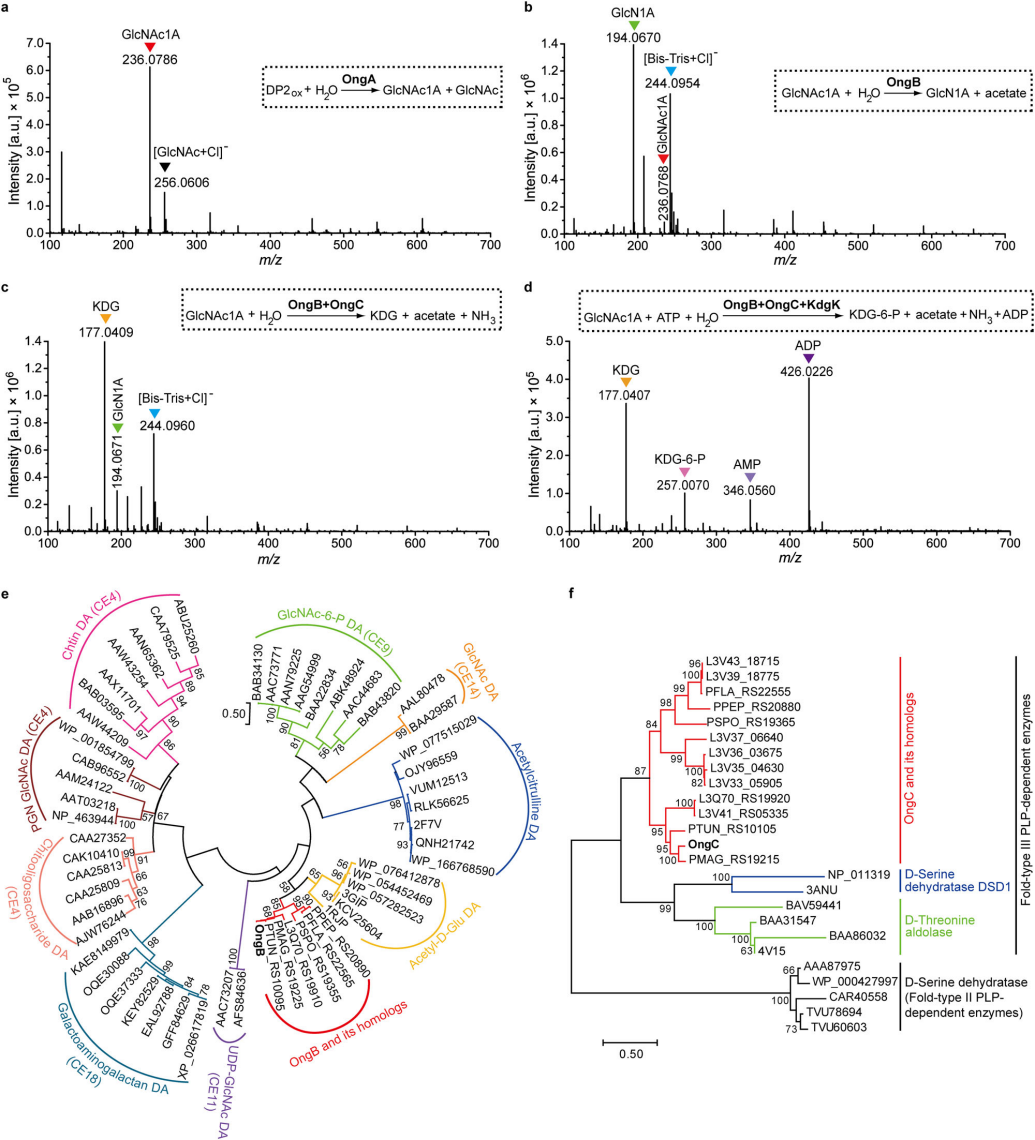
In vitro biochemical analyses of enzymes OngA, OngB, OngC and KdgK. **a** Negative-mode Q-TOF-MS spectrum of products generated by the recombinant OngA acting on GlcNAc-GlcNAc1A. Q-TOF-MS spectrum of GlcNAc-GlcNAc1A only as the control was shown in Supplementary Fig. 12a. DP2_ox_, GlcNAc-GlcNAc1A. **b** Negative-mode Q-TOF-MS spectrum of products generated by the recombinant OngB acting on GlcNAc1A. Q-TOF-MS spectrum of GlcNAc1A only as the control was shown in Supplementary Fig. 12b. **c** Negative-mode Q-TOF-MS spectrum of products generated by the recombinant enzymes, OngB and OngC, acting on GlcNAc1A. Q-TOF-MS spectrum of KDG only as the control was shown in Supplementary Fig. 12c. **d** Negative-mode Q-TOF-MS spectrum of products generated by OngB, OngC and KdgK successively acting on GlcNAc1A. Q-TOF-MS spectrum of KDG-6-P only as the control was shown in Supplementary Fig. 12d. In **a-d** all labeled MS peaks refer to [M-H]^-^ ions unless noted otherwise (e.g. [GlcNAc+Cl]^-^ ion). Theoretical masses of relevant products are listed in Supplementary Table 4. In **a-d**, the graphs show a representative MS spectrum of at least three independent replicates. **e** Maximum-likelihood tree of OngB and its homologs and other de-*N*-acetylases. All homologs to OngB (colored in red) are from the *ong* clusters of their respective bacterial strains. CE carbohydrate esterase, DA deacetylase, PGN peptidoglycan, Acetyl-D-Glu, acetyl-D-glutamate. **f** Neighbor-joining tree of OngC and its homologs and characterized pyridoxal 5-phosphate (PLP)-dependent enzymes. All homologs to OngC (colored in red) are from the *ong* clusters of their respective bacterial strains. Source data are provided as a Source Data file.

To date, enzymes responsible for the hydrolysis of oxidized chitooligosaccharides into the monosaccharides GlcNAc and GlcNAc1A are still unidentified. The *ong* cluster in strain ACAM 620 encodes a putative β-hexosaminidase, OngA, which belongs to the GH20 family and shares 32% identity to the β-*N*-acetylglucosaminidase from *Vibrio furnissii*^36^. The GH20 β-*N*-acetylglucosaminidases characterized so far all cleave terminal GlcNAc from chitooligosaccharides^37^. Phylogenetic analysis showed that OngA and its homologs encoded by the *ong* clusters are clustered as a separate group from characterized GH20 β-*N*-acetylglucosaminidases (Supplementary Fig. 9), suggesting that OngA and its homologs may be different from previously characterized β-*N*-acetylglucosaminidases in substrate specificity. Indeed, the *ongA*-deletion mutant strain (△*ongA*) was unable to grow on GlcNAc-GlcNAc1A (Fig. 3d), but still grew on GlcNAc1A (Supplementary Fig. 7a), indicating that *ongA* is essential for strain ACAM 620 to utilize GlcNAc-GlcNAc1A. Enzymatic activity analysis in vitro combined with reaction product analysis by MS demonstrated that OngA hydrolyzes GlcNAc-GlcNAc1A into GlcNAc and GlcNAc1A (Fig. 4a, Supplementary Figs. 10–12 and Supplementary Table 4). OngA was predicted to contain an N-terminal signal peptide sequence by SignalP 5.0 and to be a periplasmic protein by PSORTb 3.0 (Supplementary Table 3), suggesting that the hydrolysis of oxidized chitooligosaccharides to monosaccharides by OngA likely takes place in the periplasm.

### Transport of GlcNAc1A across inner membrane

In the *ong* cluster, a predicted inner-membrane sodium solute symporter (SSS) family protein OngIT was found, sharing 23% identity to the human sodium/glucose cotransporter SGLT1^38^. Phylogenetic analysis showed that OngIT and its homologs from the *ong* clusters of bacteria are clustered as a separate group from all characterized secondary transporters, which are more closely related to sodium/glucose cotransporters and sodium/sialic acid cotransporters than to sodium/amino acid cotransporters (Supplementary Fig. 13). The *ongIT*-deletion mutant strain (Δ*ongIT*) was unable to grow on GlcNAc1A, but its growth was fully restored by complementation of this gene (Fig. 3e), suggesting the essential role of OngIT in the uptake of GlcNAc1A in strain ACAM 620. Furthermore, compared to the wild-type strain, no difference in growth was observed for the Δ*ongIT* mutant strain on GlcNAc, GlcN or MurNAc (Supplementary Fig. 8), suggesting that OngIT is not involved in the uptake of other carbohydrates. These data indicate that OngIT specifically transports GlcNAc1A across inner membrane to the cytoplasm in strain ACAM 620.

Interestingly, (GlcNAc)_2_ resulting from the hydrolytic chitin utilization pathway in *Vibrionaceae* is transported across the inner membrane via an ABC-type transporter^39^ and GlcNAc is imported via a phosphotransferase (PTS) transporter^40^. In *S. oneidensis*, a major facilitator superfamily (MFS) permease and an acyltransferase family transporter were predicted to transport GlcNAc^11^. Different from these reported inner-membrane transporters for (GlcNAc)_2_ and GlcNAc, the inner-membrane transporter for GlcNAc1A from the oxidative chitin utilization pathway, OngIT in strain ACAM 620, is from the SSS family.

### Intracytoplasmic GlcNAc1A catabolism: OngB catalyzes the first step of GlcNAc1A catabolism by deacetylating GlcNAc1A to GlcN1A and acetate

In addition to OngA, the *ong* cluster encodes four additional enzymes, including one sugar kinase (KdgK), one RidA family protein (OngD), one putative D-aminoacylase (OngB) and one putative D-amino acid deaminase (OngC) (Fig. 3a and Supplementary Table 3). Deletion of each of these genes completely abolished the growth of strain ACAM 620 on GlcNAc1A, and complementation of their corresponding genes largely restored the bacterial growth (Fig. 3f–h and Supplementary Fig. 7b), indicating that these genes are all essential for strain ACAM 620 to utilize GlcNAc1A. Based on the functional annotation of these four genes and reported catabolic pathways for other monosaccharides^11,41,42^ and amino acids^43,44^, we hypothesize that strain ACAM 620 may adopt one of the following two ways to catabolize GlcNAc1A: 1) a way similar to the GlcNAc catabolism^10–12^, in which GlcNAc1A is phosphorylated first followed by successive deacetylation and deamination; 2) a way similar to the *N*-acetyl-D-amino acid catabolism^43,44^, in which GlcNAc1A is directly deacetylated and deaminated without activation by phosphorylation.

To elucidate how GlcNAc1A is catabolized in strain ACAM 620, we first assayed the activity of the sugar kinase KdgK in vitro against GlcNAc1A. While an excess amount of KdgK protein was added into the reaction system containing GlcNAc1A as the substrate and ATP as a cofactor for prolonged incubation (1 h) at 25 °C, only a small amount of phosphorylated GlcNAc1A was produced. Moreover, when any of the three enzymes, OngB, OngC and OngD, was added into the resultant reaction mixture, no acetate nor primary amine products were detected in the mixture, suggesting that KdgK is unlikely the first enzyme involved in GlcNAc1A degradation, and that the phosphorylated GlcNAc1A product may inhibit the activities of OngB, OngC and/or OngD. Indeed, KdgK is capable of phosphorylating 2-keto-3-deoxygluconate (KDG), a downstream metabolite in GlcNAc1A degradation (see below). Thus, we proposed that GlcNAc1A is directly deacetylated. We therefore examined the activity of OngB, OngC and OngD, respectively, against GlcNAc1A for deacetylation. Only OngB was shown to act on GlcNAc1A to produce acetate (Supplementary Fig. 14a). Further Q-TOF-MS analysis of the reaction products uncovered a product with a mass-to-charge (*m/z*) ratio of 194.0670, matching 2-(amino)-2-deoxy-D-gluconic acid (GlcN1A) (Fig. 4b, Supplementary Fig. 12 and Supplementary Table 4). Together, our data suggest that OngB initiates GlcNAc1A deacetylation to produce GlcN1A.

Among characterized enzymes, OngB is most closely related to the D-aminoacylase from *Alcaligenes faecalis*^45^, and the *N*-acetyl-D-glutamate deacetylase from *Bordetella bronchiseptica*^44^, sharing 46 and 42% sequence identities, respectively. Phylogenetic analysis suggested that OngB and its homologs from the *ong* clusters of bacteria are clustered as a separate group from all characterized de-*N*-acetylases, which are more closely related to *N*-acetyl-D-glutamate deacetylases than to carbohydrate de-*N*-acetylases (Fig. 4e). Indeed, OngB displays high substrate specificity towards GlcNAc1A, and little or no activity for GlcNAc, GlcNAc-6-P, *N*-acetyl-D-glutamate and *N*-acetyl-D-serine (Supplementary Fig. 14). Together, these data demonstrate that OngB is a functional GlcNAc1A deacetylase that catalyzes the first step of GlcNAc1A catabolism by deacetylating GlcNAc1A to GlcN1A and acetate.

### Intracytoplasmic GlcNAc1A catabolism: OngC catalyzes the second step of GlcNAc1A catabolism by deaminating GlcN1A into KDG and ammonia

To investigate which enzyme catalyzes the deamination step of GlcNAc1A catabolism, we measured the activities of OngC (a putative D-amino acid deaminase) and OngD (a RidA family protein) against GlcN1A generated in the enzymatic reaction system by OngB acting on GlcNAc1A. In the reaction system containing OngC, but not OngD, ammonia and KDG were produced (Fig. 4c, Supplementary Figs. 12 and 15 and Supplementary Table 4), indicating that OngC can deaminate GlcN1A to produce ammonia and KDG, an important metabolic intermediate in bacterial carbon metabolism^30,46,47^. Different from the well-characterized GlcN-6-P deaminase which catalyzes the deamination of GlcN-6-P with water as a co-substrate^48^, OngC performs deamination requiring no water.

Phylogenetic analysis suggested that OngC and its homologs from the *ong* clusters of bacteria form a separate clade of the Fold-Type III pyridoxal 5-phosphate (PLP)-dependent enzyme family and are distantly related to D-serine deaminases and D-threonine aldolases (Fig. 4f). Biochemical analysis showed that OngC has a broad substrate specificity and can deaminate D-serine and D-threonine in addition to GlcN1A (Supplementary Fig. 15).

### Intracytoplasmic GlcNAc1A catabolism: KdgK catalyzes the third step of GlcNAc1A catabolism by phosphorylating KDG to KDG-6-P

Due to the moderate sequence identities (~36%) between KdgK and reported KDG kinases^49^, we examined the activity of KdgK against KDG, GlcNAc and GlcNAc1A in vitro. Indeed, KdgK phosphorylated KDG efficiently but showed limited activity towards GlcNAc and GlcNAc1A (Supplementary Fig. 16). Further Q-TOF-MS analysis of the reaction products generated by OngB, OngC and KdgK successively acting on GlcNAc1A demonstrated the production of KDG-6-P (Fig. 4d, Supplementary Fig. 12 and Supplementary Table 4). These data indicate that KdgK is a functional KDG kinase, which catalyzes the third step of GlcNAc1A catabolism by phosphorylating KDG to KDG-6-P. Both Psortb 3.0 and CELLO v.2.5 were unable to predict the cellular localization of KdgK in strain ACAM 620, but strongly suggested that both OngB and OngC are cytoplasmic enzymes (Supplementary Table 3), suggesting that the GlcNAc1A catabolism catalyzed by these enzymes likely takes place in the cytoplasm of strain ACAM 620. In addition, OngD was also suggested to be essential for strain ACAM 620 to utilize GlcNAc1A based on genetic analysis in vivo (Supplementary Fig. 7b). OngD is a 127-aa small protein containing a RidA family domain. The RidA family proteins are reported to have diverse catalytic functions such as enamine/imine deaminases^50^ and translation inhibitors^51^. However, the role of OngD in GlcNAc1A catabolism is still unknown, which needs further study.

**Fig. 5:**
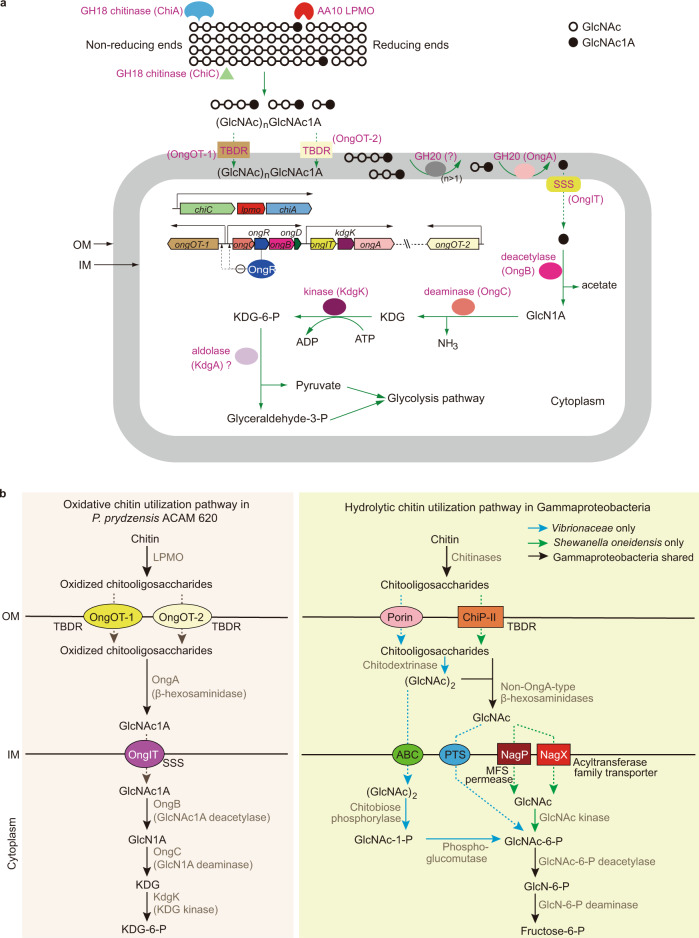
The proposed oxidative chitin utilization pathway in strain ACAM 620 and comparison with the well-characterized hydrolytic chitin utilization pathway in Gammaproteobacteria. **a** The proposed oxidative chitin utilization pathway in strain ACAM 620. Strain ACAM 620 secrets a LPMO (as well as chitinases) to cleave chitin into C1-oxidized chitooligosaccharides which are imported across the outer membrane (OM) by two TBDRs, OngOT-1 and OngOT-2. OngA in the periplasm hydrolyzes oxidized chitooligosaccharides to generate GlcNAc1A which is transported across the inner membrane (IM) by a SSS family transporter, OngIT, and converted to KDG-6-P, NH_3_ and acetate by OngB, OngC and KdgK in the cytoplasm. Enzyme/transporter symbols are colored according to the colors of their genes. Green solid arrows denote enzymatic reactions, and green dotted arrows denote transport. Black arrows represent the transcriptional directions of the *cdc* cluster as one operon and predicted operons of the *ong* cluster. The minus sign surrounded by a circle represents negative regulation. **b** Comparison of the proposed oxidative chitin utilization pathway in strain ACAM 620 and reported hydrolytic chitin utilization pathway in other Gammaproteobacteria. In the hydrolytic chitin utilization pathway, chitinases degrade chitin into chitooligosaccharides which are imported across OM by a specific porin in *Vibrionaceae*^8,9,35^ or a predicted TBDR in *Shewanella oneidensis*^11^. Non-OngA-type β-hexosaminidases in the periplasm hydrolyze chitooligosaccharides into GlcNAc which is transported into the cytoplasm by non-SSS-type transporters and converted to fructose-6-P, NH_3_ and acetate by a GlcNAc kinase (or a PTS), a GlcNAc-6-P deacetylase and a GlcN-6-P deaminase successively. Enzymes involved in oxidative and hydrolytic chitin utilization pathways are shown in grey. Solid arrows denote enzymatic reactions, and dotted arrows denote transport.

For amino sugars including GlcNAc, *N*-acetyl-D-galactosamine, *N*-acetyl-D-mannosamine and MurNAc and other monosaccharides such as D-glucose, D-gluconate, D-galactose and D-ribose, the catabolism of all these sugars is initiated by phosphorylation^11,41,42,52,53^. In contrast, our results indicated that the catabolism of GlcNAc1A is initiated by deacetylation and deamination directly without activation by phosphorylation (Figs. 1 and 5a). In some bacteria, catabolism of *N*-acetyl-D-serine and *N*-acetyl-D-threonine starts with deacetylation followed by deamination and formation of α-keto acids^44,54^. GlcNAc1A is more similar to *N*-acetyl-D-amino acids than to GlcNAc in terms of chemical structure and charge. Correspondingly, enzymes for catalyzing the deacetylation and deamination steps of the GlcNAc1A catabolism are more phylogenetically closely related to those involved in D-amino acid catabolism than those in GlcNAc catabolism (Fig. 4e and f). Moreover, GlcNAc1A is deacetylated and deaminated to generate KDG with a carbonyl group adjacent to its carboxylic group, which is also a kind of α-keto acids. Therefore, GlcNAc1A is catabolized in strain ACAM 620 via a pathway akin to the catabolism of D-amino acids rather than other monosaccharides.

### OngR is a regulatory protein in GlcNAc1A catabolism

The *ong* cluster encodes a putative MurR/RpiR family transcriptional regulator, OngR, which displays 24% identity to the repressor RpiR of D-allose catabolism in *E. coli*^55^. Compared to the wild-type strain, the *ongR*-deletion mutant strain (Δ*ongR*) exhibited better growth on GlcNAc1A (Fig. 3i), indicating that OngR functions as a repressor. Transcriptomic analysis on cells cultivated on GlcNAc1A suggested that genes from the *ong* cluster were co-transcribed as three discrete operons, including 1) *ongOT-1*, 2) *ongCRBD*, and 3) *ongIT*-*kdgK*-*ongA*, whereas genes from the *cdc* cluster formed an operon. To reveal which of these operons are regulated by OngR, the transcriptional levels of the genes in the *ong* cluster, as well as *ongOT-2* and *lpmo* in the Δ*ongR* mutant strain were quantified via real-time quantitative PCR (RT-qPCR) and compared to those of the wild-type strain in response to monosaccharides including GlcNAc1A, GlcNAc and glucose. No significant differences were observed for the transcriptional levels of *ongIT*, *kdgk*, *ongA*, *ongOT-2* and *lpmo* in both the wild-type and mutant strains on these substrates (Fig. 3j and Supplementary Fig. 17), indicating that the transcription of the *ongOT-2*, *cdc* and *ongIT*-*kdgK*-*ongA* operons is not regulated by OngR. However, the transcriptional levels of four genes including *ongOT-1*, *ongC*, *ongB* and *ongD* were strongly repressed in the wild-type strain from the lag phase to the exponential phase on both GlcNAc and glucose, but robustly induced by GlcNAc1A during the exponential phase (Fig. 3j and Supplementary Fig. 17), indicating that the transcription of the *ongOT-1* and *ongCRBD* operons is negatively regulated by OngR. Moreover, in the Δ*ongR* mutant strain, enhanced transcriptional expression of *ongOT-1*, *ongC*, *ongB* and *ongD* occurred regardless of the presence or absence of GlcNAc1A (Fig. 3j and Supplementary Fig. 17), further supporting that OngR regulates the GlcNAc1A catabolism in strain ACAM 620 by repressing the transcription of the *ongOT-1* and *ongCRBD* operons. This repressor-dependent regulation of key genes involved in oxidative chitin degradation is therefore significantly different from hydrolytic chitin degradation in *Vibrio cholerae* and *Pseudoalteromonas piscicida* whereby bacterial two-component systems play an essential role in modulating the expression of chitinases^39,56^.

### The characterized oxidative chitin utilization pathway in strain ACAM 620 differs from the well-established hydrolytic chitin utilization pathway

Based on the above results, we propose the oxidative chitin utilization pathway in strain ACAM 620, involving the LPMO and the *ong* cluster. This pathway involves five steps: 1) extracellular breakdown of chitin polymer into C1-oxidized chitooligosaccharides by a LPMO as well as chitinases (ChiA and ChiC), 2) transport of the oxidized chitooligosaccharides across the outer membrane by specific TBDRs, OngOT-1 and OngOT-2, 3) hydrolysis of the oxidized chitooligosaccharides into GlcNAc1A and GlcNAc by OngA in the periplasm, 4) transport of GlcNAc1A across the inner membrane by a SSS family transporter, OngIT, and 5) processive catabolism of GlcNAc1A to KDG-6-P, acetate and NH_3_ by OngB, OngC and KdgK via a pathway akin to the D-amino acid catabolism (Figs. 1 and 5a). We hypothesize that KDG-6-P is further metabolized by potential KDG-6-P aldolases present in strain ACAM 620 (Supplementary Table 3) into pyruvate and glyceraldehyde-3-P to enter the glycolysis pathway. In addition, OngR regulates the oxidative chitin utilization pathway in strain ACAM 620 mainly through repressing the transcription of the *ongOT-1* and *ongCRBD* operons.

In the well-characterized hydrolytic chitin utilization pathway (Fig. 5b), chitinases degrade chitin into chitooligosaccharides which are imported across the outer membrane by a specific porin in the *Vibrionaceae*^35^ or a predicted TBDR in *S. oneidensis*^11^. Non-OngA-type β-hexosaminidases in the periplasm hydrolyze chitooligosaccharides into GlcNAc, which is transported into the cytoplasm by non-SSS-type transporters and converted to fructose-6-P, NH_3_ and acetate by GlcNAc kinase (or PTS), GlcNAc-6-P deacetylase and GlcN-6-P deaminase^8,9,11^. Moreover, OngR involved in oxidative chitin utilization in strain ACAM 620 is not involved in hydrolytic chitin utilization. Therefore, the oxidative chitin utilization pathway in strain ACAM 620 is significantly different from the hydrolytic chitin utilization pathway in enzymes, transporters and regulators.

It is likely that the complete utilization of marine chitin by strain ACAM 620 depends on a synergistic action of oxidative and hydrolytic degradation. The efficient degradation of natural crystalline chitin by the LPMO from strain ACAM 620 (Fig. 2d and Supplementary Fig. 4) and the only reported marine LPMO from *Aliivibrio salmonicida* LFI1238^21^ suggests the key role of oxidation degradation in the initial degradation of marine chitin particles, which facilitates further chitin degradation by chitinases. Moreover, the reduced growth of the mutants of strain ACAM 620, including Δ*lpmo*, Δ*ongOT-1/*Δ*ongOT-2* and Δ*ongB*, on crystalline α- and β-chitin (Supplementary Fig. 18) also indicates the important role of oxidative degradation involving the LPMO and the *ong* cluster in chitin degradation. However, considering that the LPMO protein abundance in the secretome of strain ACAM 620 is much lower than that of chitinases (Fig. 2c and Supplementary Data 1), the relative contribution of oxidative degradation versus hydrolytic degradation of marine chitin warrants further investigation.

### The oxidative chitin utilization pathway is found in many marine bacteria

**Fig. 6:**
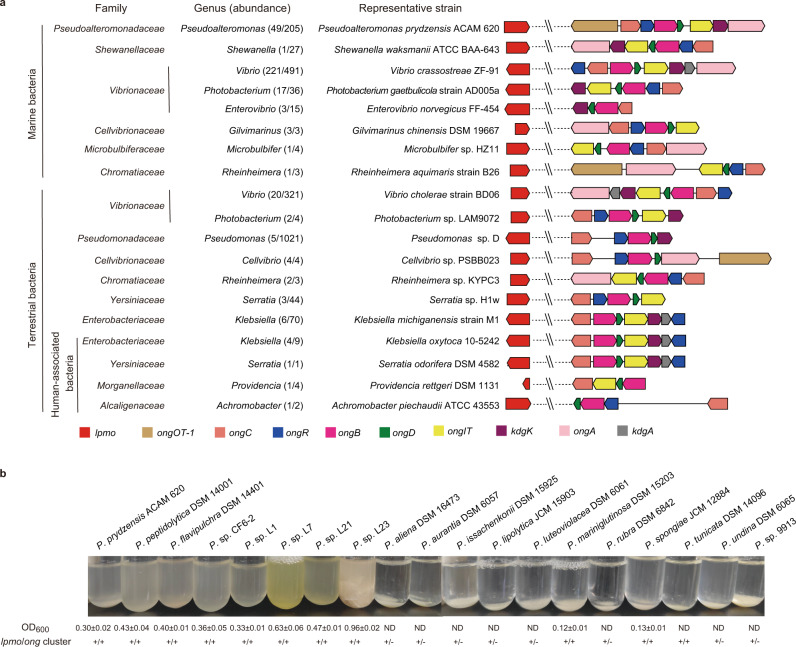
Distribution and ecological function of the oxidative chitin utilization pathway in bacteria. **a** Distribution of the oxidative chitin utilization pathway in marine and terrestrial bacterial isolates. Except for terrestrial bacterium *Achromobacter piechaudii* ATCC 43553 belonging to Betaproteobacteria, all other bacterial strains with the complete oxidative chitin utilization pathway are from Gammaproteobacteria. For some representative strains containing more than one *lpmo* genes, only one *lpmo* gene was shown. **b** Comparison of the chitin-degrading abilities and related chitin-degrading genes of *Pseudoalteromonas* strains. Strains were cultivated in the minimal medium supplemented with 0.2% (w/v) shrimp shell α-chitin as the sole carbon source at 25 °C for 7 days. No bacterial growth was detectable for nine *Pseudoalteromonas* strains including *P. aliena* DSM 16473, *P. aurantia* DSM 6057, *P. issachenkonii* DSM 15925, *P. lipolytica* JCM 15903, *P. luteoviolacea* DSM 6061, *P. rubra* DSM 6842, *P. tunicata* DSM 14096, *P. undina* DSM 6065 and *P*. sp. SM9913, even though they were cultivated on α-chitin for 14 days at 25 °C. ND, undetectable growth; +, presence; -, absence. Growth data are presented as mean ± SD (*n* = 3 independent experiments). Source data are provided as a Source Data file.

To better understand the ecological significance of the chitin oxidative metabolism in marine bacteria, the genes from the *ong* cluster as well as *lpmo* of strain ACAM 620 were searched against the genomes of marine isolates in the RefSeq database at NCBI. As a result, 298 out of 2,455 marine bacteria were found to harbor both the *ong* cluster and *lpmo* (Fig. 6a and Supplementary Data 2). These bacteria were isolated from a variety of environmental samples, including seawater (236), sediments (12), marine invertebrates/vertebrates (34), marine algae (8), and other (8) (Supplementary Data 2). All strains belong to Gammaproteobacteria, particularly from genera *Vibrio* (221), *Pseudoalteromonas* (49), and *Photobacterium* (17) (Fig. 6a and Supplementary Data 2). The *ong* cluster and *lpmo* are also present in terrestrial Gammaproteobacteria mainly affiliated with genera *Vibrio*, *Klebsiella*, *Pseudomonas* and *Cellvibrio* (Fig. 6a and Supplementary Data 3 and 4). Except for the *ongOT-1* gene, all other genes from the *ong* cluster are highly conserved in bacteria (Fig. 6a), suggesting the conservation of the oxidative chitin utilization pathway in bacteria. Notably, the *ong* clusters from genera *Vibrio*, *Klebsiella* and *Serratia* carry an additional *kdgA* gene encoding a putative KDG-6-P aldolase (Fig. 6a), supporting our hypothesis that the intermediate KDG-6-P may be further metabolized by KDG-6-P aldolases to pyruvate and glyceraldehyde-3-P to enter the glycolysis pathway.

Moreover, strain ACAM 620 and other marine *Pseudoalteromonas* strains containing both the *ong* cluster and *lpmo* tend to have stronger crystalline chitin-degrading abilities than those without the *ong* cluster (Fig. 6b and Supplementary Table 1), suggesting that the presence of a complete oxidative chitin utilization pathway facilitates bacterial degradation of crystalline chitin. Marine *Vibrio* strains containing both the *ong* cluster and *lpmo* also exhibit crystalline chitin-degrading abilities (Supplementary Table 5). The degradation of marine chitin particles is reported to be limited by the number of chitinolytic bacteria colonizing the particle surface, especially by those with high chitin-degrading activities^57^. The bacterial groups with a complete oxidative chitin utilization pathway are therefore likely the key degraders in the initial degradation of marine chitin particles. In addition, metagenomic analysis against the Polar marine reference gene catalog (PM-RGC)^58^ and Tara Oceans metagenomes^59^ showed that AA10 LPMO-like and OngB-like sequences are present in many marine Gammaproteobacteria (Supplementary Figs. 1 and 19), supporting that the oxidative chitin utilization pathway extensively exists in marine Gammaproteobacteria.

In summary, using transcriptomic, proteomic, genetic and biochemical analyses, the complete oxidative chitin utilization pathway in *P. prydzensis* ACAM 620 is revealed in this study. This pathway, which exists in many marine Gammaproteobacteria, enhances bacterial degradation of crystalline chitin, highlighting the importance of these bacteria in the initial degradation of crystalline chitin in marine environments.

## Methods

### Marine chitinolytic *Pseudoalteromonas* and *Vibrio* strains

A total of 30 marine chitinolytic strains were used (Supplementary Tables 1 and 5), including 13 type strains of *Pseudoalteromonas* purchased from Deutsche Sammlung von Mikroorganismen and Zelkulturen (DSMZ) and Japan Collection of Microorganisms (JCM) and 17 non-type strains (6 *Pseudoalteromonas* strains and 11 *Vibrio* strains) isolated by our laboratory. To determine the chitin-degrading ability, all strains were cultivated at 25 °C and 180 rpm for 4-14 days in the liquid medium containing the minimal medium (0.05% (w/v) NH_4_Cl, 3% (w/v) NaCl, 0.3% (w/v) MgCl_2_·6H_2_O, 0.2% (w/v) K_2_SO_4_, 0.02% (w/v) K_2_HPO_4_, 0.001% (w/v) CaCl_2_, 0.0006% (w/v) FeCl_3_·6H_2_O, 0.0005% (w/v) NaMoO_4_·7H_2_O, 0.0004% (w/v) CuCl_2_·2H_2_O and 0.6% (w/v) Tris (pH 7.8)) supplemented with α-chitin powder from shrimp shell (Sigma, USA), β-chitin flakes from squid pen (Hubei Yuancheng Saichuang Technology Co., Ltd., China) or colloidal chitin made from shrimp shell α-chitin at a concentration of 0.2% (w/v) as the sole carbon source.

### Utilization of GlcNAc1A and other carbon sources by *Pseudoalteromonas* and *Vibrio* strains

To test the growth of marine *Pseudoalteromonas* spp. and *Vibrio* spp. on different carbon sources, strains were inoculated into the minimal medium supplemented with GlcNAc1A, GlcNAc, D-gluconate or other substrates at a concentration of 0.2% (w/v) as the sole carbon source and cultivated at 25 °C and 180 rpm. The growth of strains was measured by detecting the OD_600_ of the cultures at different time points using a spectrophotometer V-550 (Jasco Corporation, Japan).

### Genome analysis

Genomic DNA of all non-type strains of chitinolytic *Pseudoalteromonas* and *Vibrio* but strain SM9913 was extracted using the PowerMax® Soil DNA Isolation Kit (MO BIO laboratories, Inc., USA) according to the manufacturer’s instructions. Genome sequencing was performed using the Illumina HiSeq2000 platform at BGI-Shenzhen, China. Raw reads were trimmed by Trimmomatic version 0.36^60^. The obtained clean reads were then assembled to scaffolds using SPAdes version 3.11.1^61^ and the intrascaffold gaps were closed by GapCloser version 1.1 (https://sourceforge.net/projects/soapdenovo2/files/GapCloser/). The resultant assemblies were annotated by the RAST server^62^. The genome sequences of strain SM9913^63^ and type *Pseudoalteromonas* strains^64^ have been deposited in the NCBI GenBank by our lab previously. Genes related to oxidative degradation of chitin were searched against the genomes of chitinolytic *Pseudoalteromonas* and *Vibrio*. Cellular location of proteins was predicted by PSORTb v3.0^65^ and CELLO v.2.5^66^ combined with SignalP 5.0^67^.

### Secretome analysis

Pre-cultures of strain ACAM 620 in marine broth 2216 were rinsed with sterile artificial seawater three times prior to inoculation. Strain ACAM 620 was then cultivated in the minimal medium supplemented with 0.5% (w/v) colloidal chitin at 25 °C until approximately half of chitinous substrate was degraded. The culture was pelleted by centrifugation and the supernatant was collected. Label-free quantitative LC-MS/MS was performed to study the soluble extracellular proteins of strain ACAM 620 with two biological replicates. The extracellular proteins were precipitated in cold acetone solution containing 10 mM dithiothreitol at −20 °C overnight. The precipitates were harvested by centrifugation for 20 min at 21,000 × *g* and 4 °C, washed twice by ice-cold acetone solution, and then dried. Pierce BCA Protein Assay Kit (Thermo Scientific, USA) was used to determine the protein concentrations of samples. The protein sample (100 μg) was digested using 2 μg trypsin (Promega, Madison, WI) at 37 °C overnight. Peptides in the sample were trapped and desalted on the C18 column using 80% acetonitrile/H_2_O containing 0.1% trifluoroethanoicacid as the eluent. Peptides were analyzed by online nanospray LC-MS/MS on an Orbitrap Fusion^TM^ Lumos^TM^ coupled to EASY-nLC 1200 system (Thermo Scientific, USA). Protein searches against a target-decoy protein sequence database of strain ACAM 620 were performed using MaxQuant^68^ with a peptide level FDR (false discovery rate) set to 0.01. Proteins were considered detected only when they were present in both replicates. Abundance was calculated based on the proportion of the iBAQ (intensity-based absolute quantification) of a protein in the sum of iBAQs of all proteins in the same sample and indicated by relative iBAQ (riBAQ) values. To uncover chitinolytic enzymes in the secretome, all detected proteins belonging to carbohydrate-active enzymes were determined according to dbCAN^69^ and Pfam^70^ analyses. Cellular location of identified proteins was predicted according to PSORTb v3.0^65^ and CELLO v.2.5^66^ combined with SignalP 5.0^67^.

### Transcriptome analysis

Pre-cultures of strain ACAM 620 in the minimal medium supplemented with 0.2% (w/v) glucose were rinsed with the minimal medium three times prior to inoculation. Strain ACAM 620 was then cultivated in the minimal medium supplemented with either 0.2% (w/v) GlcNAc1A or 0.2% (w/v) glucose to reach early- and mid-exponential phases. Cells were collected by centrifugation, and the total RNA was extracted using the RNeasy Mini Kit (Qiagen, Germany). Sample processing, library preparation and sequencing were performed by Majorbio Co. Ltd. (Shanghai, China), and the reads were mapped to the genome of strain ACAM 620. Gene expression levels were calculated using Rockhopper version 2.0.3^71^ and normalized using the RPKM (reads per kilobases per million mapped reads) method, and the RPKM value of each gene was used to compare levels of expression under different conditions. A gene was considered to be differentially regulated when it showed a > 2-fold change in expression and displayed an FDR-adjusted *P* value of <0.05.

### Construction of mutant and complementary strains

Using vectors pK18mobsacB-Ery^72^ and pEV^73^ for gene knockout and gene complementation, respectively, the knockout mutants of strain ACAM 620, Δ*lpmo*, Δ*ongA*, Δ*ongB*, Δ*ongC*, Δ*ongD*, Δ*kdgK*, Δ*ongOT-1*, Δ*ongOT-2*, Δ*ongOT-1/*Δ*ongOT-2*, Δ*ongIT* and Δ*ongR*, as well as their complementary strains, Δ*ongA*/pEV*ongA*, Δ*ongB*/pEV*ongB*, Δ*ongC*/pEV*ongC*, Δ*ongD*/pEV*ongD*, Δ*kdgK*/pEV*kdgK*, Δ*ongOT-1/*Δ*ongOT-2/*pEV*ongOT-1*, Δ*ongOT-1/*Δ*ongOT-2/*pEV*ongOT-2*, Δ*ongIT*/pEV*ongIT* and Δ*ongR*/pEV*ongR*, were constructed. The empty plasmid pEV containing an ampicillin resistance gene and a chloramphenicol resistance gene was also mobilized into the knockout mutants of strain ACAM 620 to construct mutants, Δ*ongA*/pEV, Δ*ongB*/pEV, Δ*ongC*/pEV, Δ*ongD*/pEV, Δ*kdgK*/pEV, Δ*ongOT-1/*Δ*ongOT-2/*pEV, Δ*ongIT*/pEV and Δ*ongR*/pEV, as the negative control. All mutants were verified via DNA sequencing. Bacterial strains, plasmids and primers used for genetic manipulations of strain ACAM 620 are listed in Supplementary Tables 6 and 7 and Supplementary Data 5. The growth curves of wild-type strain and its mutant and complementary strains were compared in the minimal medium supplemented with 10 mM soluble substrates (GlcNAc1A, GlcNAc-GlcNAc1A or other soluble substrates) or 0.2% (w/v) crystalline substrates (α- or β-chitin) as the sole carbon source. For the cultivation of mutant strains carrying an empty pEV plasmid and complementary strains, 100 μg/ml ampicillin and 25 μg/ml chloramphenicol were supplemented in the medium.

### RT-qPCR analysis

Pre-cultures of strain ACAM 620 and its mutant strain Δ*ongR* in the minimal medium supplemented with 0.2% (w/v) glucose were rinsed with the minimal medium three times prior to inoculation. Strain ACAM 620 and its mutant strain Δ*ongR* were then cultivated in the minimal medium supplemented with 0.2% (w/v) GlcNAc1A, 0.2% (w/v) Glucose or 0.2% (w/v) GlcNAc at 25 °C and 180 rpm. Strain ACAM 620 was also cultivated in the minimal medium supplemented with 0.5% (w/v) colloidal chitin. Cells were collected from each culture at the exponential phase as well as pre-cultures. Total RNA was extracted using the RNeasy Mini Kit (Qiagen, Germany). Reverse transcription was performed by using TransScript First-Strand cDNA Synthesis SuperMix (TransGen Biotech, China). The RT-qPCR reaction was performed on the LightCycler® 480 (Roche, Switzerland) and SYBR green fluorescence (Takara, Japan) was used for detection. The relative expression of the target gene was normalized to the reference gene of *rpoD* that was not regulated by different carbon sources. Each sample for RT-qPCR was performed in triplicate. Primers used for RT-qPCR are listed in Supplementary Data 5.

### Expression and purification of recombinant proteins

By using gene-specific primers (Supplementary Data 5), the LPMO-encoding gene without the signal peptide sequence was amplified from the genomic DNA of strain ACAM 620, and the amplified fragments were ligated into the vector pET22b between the NcoI and XhoI sites. Genes *chiA*, *chiC* and *ongA* without the signal peptide sequences and the full-length genes *ongB*, *ongC*, *ongD* and *kdgK* were amplified from the genomic DNA of strain ACAM 620 respectively, and the amplified fragments were ligated into the vector pET22b between the NdeI and XhoI sites. The constructed plasmids were then transformed into *E. coli* BL21 (DE3). The recombinant *E. coli* strains were cultured at 37 °C in Lysogeny broth medium to an OD_600_ of 0.6 to 1.0, and then cultivated at 18 °C for 16 hours with 0.5 mM isopropyl-β-D-thiogalactopyranoside (IPTG) as an inducer. Cells were collected by centrifugation. Recombinant LPMO without a C-terminal His tag was extracted from the periplasmic fraction of cells by cold osmotic shock^74^ and purified by anion exchange chromatography using a 5 ml HiTrap DEAE EF column (GE Healthcare, Sweden). Other recombinant proteins with a C-terminal His tag were extracted from the cellular fraction of cells and purified with Ni-nitrilotriacetic acid (NTA) resin (Qiagen, USA). Purified proteins were desalted with PD-10 desalting columns (GE Healthcare, Sweden) and protein concentrations were determined by a Pierce BCA Protein Assay Kit (Thermo Scientific, USA).

### Enzyme assays and identification of products

The LPMO activity was determined using the method described by Loose et al. (2014)^15^. Briefly, LPMO was saturated with copper by incubation with a 3-fold molar excess of Cu(II)SO_4_ at room temperature for 30 min. Excess copper was removed by desalting the protein solution using PD-10 desalting columns (GE Healthcare, Sweden). Standard reaction system contained 0.2% (w/v) crystalline substrates (α-chitin, β-chitin or cellulose), 1 µM of Cu^2+^ saturated LPMO, 1 mM AscA and 20 mM Tris-HCl (pH 7.5). Reactions were conducted at 25 °C for 48 h and AscA was added to the reaction systems to reach a final concentration of 1 mM at an interval of 24 h. The same reaction system without the reductant or LPMO was used as a control. The resulting mixture was centrifugated and the supernatant was analyzed with Q-TOF-MS (Bruker Impact HD, Germany) for *m/z* determination. For MS analysis, the following operating parameters were used: drying N_2_ gas flow rate, 4 l/min; temperature, 180 °C; nebulizer pressure, 6 psi; capillary, 4,500 V; and End Plate Offset, 500 V. The acquisition mass range used was from *m/z* 395 to 1,500 in positive ion mode. MS data were collected using OtofControl version 3.4 (Bruker Daltonics, Germany) and processed by DataAnalysis version 4.2 (Bruker Daltonics, Germany). The activity of the LPMO against α-chitin was also assayed in the presence of 100 µM H_2_O_2_, and the concentration of AscA used for this assay was 10 µM.

GlcNAc-GlcNAc1A was prepared from chitobiose by using the chitooligosaccharide oxidase ChitO from *Fusarium graminearum*^75^ and purified by gel filtration chromatography on a Superdex Peptide 10/300 GL column (GE Healthcare, Sweden) using 0.2 M NH_4_HCO_3_ as the running buffer. Purity of the eluted GlcNAc-GlcNAc1A was examined by Q-TOF-MS. The β-hexosaminidase activity of OngA was assayed by adding 10 μΜ OngA to 50 mM GlcNAc-GlcNAc1A in 5 mM PBS buffer (pH 7.5). The mixture was incubated at 25 °C for 12 h. The same reaction system without OngA was used as a control. The reaction products were separated on the Superdex Peptide 10/300 GL column and further identified by Q-TOF-MS from *m/z* 50 to 1,500 in negative ion mode.

The deacetylase activity was assayed by incubating 5 μΜ protein with 25 mM substrate in 10 mM Bis-Tris-HCl (pH 7.5) at 25 °C for 30 min. Substrate specificity assays were performed with GlcNAc1A, GlcNAc, GlcNAc-6-P, *N*-acetylmannosamine, *N*-acetylneuraminic acid and *N*-acetyl-D-amino acids. The same reaction system without enzyme was used as a control. The production of acetate in the reaction mixture was determined with an Acetic Acid (ACS Analyser Format) Assay Kit (Megazyme, Ireland). One unit of enzyme (U) is defined as the amount of enzyme required to release 1 μmol of acetate per minute. The reaction products were further identified by Q-TOF-MS from *m/z* 50 to 1,500 in negative ion mode.

GlcN1A was prepared by incubating 10 μΜ OngB with 10 mM GlcNAc1A in 10 mM Bis-Tris-HCl (pH 7.5) at 25 °C for 4 h. The deaminase activity was assayed by incubating 0.5 μΜ protein with 10 mM substrate in 10 mM Bis-Tris-HCl (pH 7.5) at 25 °C for 30 min. Substrate specificity assays were performed with GlcN1A, D-glucosamine, D-galactosamine, D-mannosamine and D-amino acids. The same reaction system without enzyme was used as a control. The production of ammonia in the reaction mixture was determined with an AMMONIA (Rapid) ASSAY PROCEDURE (Megazyme, Ireland). One unit of enzyme (U) is defined as the amount of enzyme required to release 1 μmol of ammonia per minute. The reaction products were further identified by Q-TOF-MS from *m/z* 50 to 1,500 in negative ion mode.

The kinase activity of KdgK was determined by measuring the amount of ADP products using an enzyme-coupled spectrophotometric assay^11^. Standard reaction contained 10 mM Tris-HCl (pH 7.5), 0.05 μΜ KdgK, 1 mM substrate, 10 mM MgSO_4_, 1.2 mM ATP, 1.2 mM phosphoenolpyruvate, 0.3 mM NADH, 1.2 units of pyruvate kinase and 1.2 units of lactate dehydrogenase. After incubation at 25 °C for 5 min, the conversion of ATP to ADP was enzymatically coupled to the oxidation of NADH to NAD^+^ and monitored at 340 nm. Substrate specificity assays were performed with KDG, GlcNAc1A and GlcNAc. The same reaction system without KdgK was used as a control. One unit of enzyme (U) is defined as the amount of enzyme required to produce 1 μmol NAD^+^ per minute. The reaction products were further identified by Q-TOF-MS from *m/z* 50 to 1,500 in negative ion mode. In addition, the chitinase activities of ChiA and ChiC against 4-methylumbelliferyl-(GlcNAc)_2_, colloidal chitin and crystalline α-chitin and their acting modes were assayed at 25 °C and pH 6.0.

### Distribution of LPMO and the *ong* cluster in marine metagenomes and bacterial genomes

Using the LPMOs (catalytic domains only) of marine strain ACAM 620 and terrestrial *Serratia marcescens*^13^ as queries, BLASTP analysis was performed using a stringency of 20% identity, a coverage of 50% and a cutoff value of 10^−5^ against the PM-RGC^58^ and Tara Oceans metagenomes^59^. To identify the *ong* cluster in marine environments, BLASTP analysis against marine metagenomes was performed using the OngB of strain ACAM 620 as the query, hits with *E*-value <10^−5^ and > 45% sequence identity were probed. The abundance of bacteria harboring LPMO or OngB in metagenomes was normalized to the total microbial community using the single-copy housekeeping gene *recA*. RecA sequences in metagenomes were retrieved by using a cutoff value of 10^−40^. Marine and terrestrial bacterial genomes were downloaded from the RefSeq database at NCBI and screened for LPMO and the *ong* cluster. The *ong* clusters in bacterial isolates were detected based on the presence of at least four co-occurring homologous genes (with an *E*-value of <10^−5^ and a sequence identity of > 30%) to those from the *ong* cluster of strain ACAM 620. In total, 2,455 marine and 12,220 terrestrial bacterial genomes (including 938 human-associated bacterial genomes) were used for analysis.

### Phylogenetic analyses of LPMO, OngA, OngB, OngC and OngIT

All LPMOs were obtained from the *cdc* clusters of chitinolytic *Pseudoalteromonas* strains assayed in this study. All homologs to OngA, OngB, OngC and OngIT were obtained from the *ong* clusters of chitinolytic *Pseudoalteromonas* and/or *Vibrio* strains assayed in this study. *Pseudoalteromonas* LPMOs and characterized chitin-active LPMOs (predicted catalytic domains only), OngA and characterized GH20 enzymes, OngB and characterized de-*N*-acetylases including *N*-acetyl-D-glutamate deacetylases and carbohydrate de-*N*-acetylases, OngC and characterized PLP-dependent enzymes, and OngIT and characterized secondary transporters were aligned by MUSCLE^76^ and visualized using Molecular Evolutionary Genetics Analysis version 7.0 (MEGA7)^77^, respectively.

### Statistics and reproducibility

Data analyses were carried out using Microsoft Excel 2016, OriginPro 8.5 and OriginPro 2022b. All data shown are means ± SD. For Q-TOF-MS analysis, SDS-PAGE analysis and gel filtration chromatography analysis, similar results were obtained from three independent experiments. Detailed data analyses are described in the text.

### Reporting summary

Further information on research design is available in the Nature Research Reporting Summary linked to this article.

## Supplementary information

Supplementary Information

Description of Additional Supplementary Files

Supplementary Dataset 1

Supplementary Dataset 2

Supplementary Dataset 3

Supplementary Dataset 4

Supplementary Dataset 5

Reporting Summary

## Data Availability

All data supporting the findings of this study are available within the paper (and its Supplementary Information files). Genome sequences obtained in this study have been deposited in GenBank/DDBJ under accession numbers CP091442 (https://www.ncbi.nlm.nih.gov/assembly/GCF_021729425.1/), CP091443 (https://www.ncbi.nlm.nih.gov/assembly/GCF_021729425.1/), JAKKCI000000000 (https://www.ncbi.nlm.nih.gov/assembly/GCF_021726015.1/), JAKKCJ000000000 (https://www.ncbi.nlm.nih.gov/assembly/GCF_021726515.1/), JAKKCK000000000 (https://www.ncbi.nlm.nih.gov/assembly/GCF_021725735.1/), JAKKCL000000000 (https://www.ncbi.nlm.nih.gov/assembly/GCF_021728175.1/), JAKKCM000000000 (https://www.ncbi.nlm.nih.gov/assembly/GCF_021728395.1/), JAKKCN000000000 (https://www.ncbi.nlm.nih.gov/assembly/GCF_021728155.1/), JAKKCO000000000 (https://www.ncbi.nlm.nih.gov/assembly/GCF_021728355.1/), JAKKCP000000000 (https://www.ncbi.nlm.nih.gov/assembly/GCF_021727995.1/), JAKKCQ000000000 (https://www.ncbi.nlm.nih.gov/assembly/GCF_021728315.1/), JAKKCR000000000 (https://www.ncbi.nlm.nih.gov/assembly/GCF_021728015.1/), JAKKCS000000000 (https://www.ncbi.nlm.nih.gov/assembly/GCF_021728075.1/), JAKKCT000000000 (https://www.ncbi.nlm.nih.gov/assembly/GCF_021728095.1/), JAKKCU000000000 (https://www.ncbi.nlm.nih.gov/assembly/GCF_021728055.1/), JAKKCV000000000 (https://www.ncbi.nlm.nih.gov/assembly/GCF_021728035.1/), and JAKKCW000000000 (https://www.ncbi.nlm.nih.gov/assembly/GCF_021728455.1/). All the RNA-seq read data have been deposited in NCBI’s sequence read archive (SRA) under project accession number PRJNA196223. The mass spectrometry proteomics data have been deposited in the ProteomeXchange Consortium via the PRIDE with identifier PXD031176. A reporting summary for this article is available as a Supplementary Information file. Source data are provided with this paper.

## Source data

Source Data
