# Supplementary material for: A pathway for chitin oxidation in marine bacteria
Source: Nat Commun. 2022 Oct 6;13:5899. doi: 10.1038/s41467-022-33566-5 (PMC9537276; doi:10.1038/s41467-022-33566-5)
Supplement: Supplementary file 1 — Supplementary Information [file 41467_2022_33566_MOESM1_ESM.pdf]

Supplementary Information

A pathway for chitin oxidation in marine bacteria

Wen-Xin Jiang[1,2]†, Ping-Yi Li[1,3]†*, Xiu-Lan Chen[1,3], Yi-Shuo Zhang[1], Jing-Ping Wang[1], Yan-Jun Wang[1], Qi Sheng[1], Zhong-Zhi Sun[1], Qi-Long Qin[1,3], Xue-Bing Ren[1], Peng Wang[2,3], Xiao-Yan Song[1], Yin Chen[2,4], Yu-Zhong Zhang[2,3,5]*

[1]State Key Laboratory of Microbial Technology, Shandong University, Qingdao, China

[2]College of Marine Life Sciences, Ocean University of China, Qingdao, China

[3]Laboratory for Marine Biology and Biotechnology, Pilot National Laboratory for Marine Science and Technology, Qingdao, China

[4]School of Life Sciences, University of Warwick, Coventry, CV4 7AL, United Kingdom

[5]Marine Biotechnology Research Center, State Key Laboratory of Microbial Technology, Shandong University, Qingdao, China

† Wen-Xin Jiang and Ping-Yi Li contributed equally to this work.

* Corresponding author: Ping-Yi Li, lipingyipeace@sdu.edu.cn; Yu-Zhong Zhang, zhangyz@sdu.edu.cn

Supplementary Figures

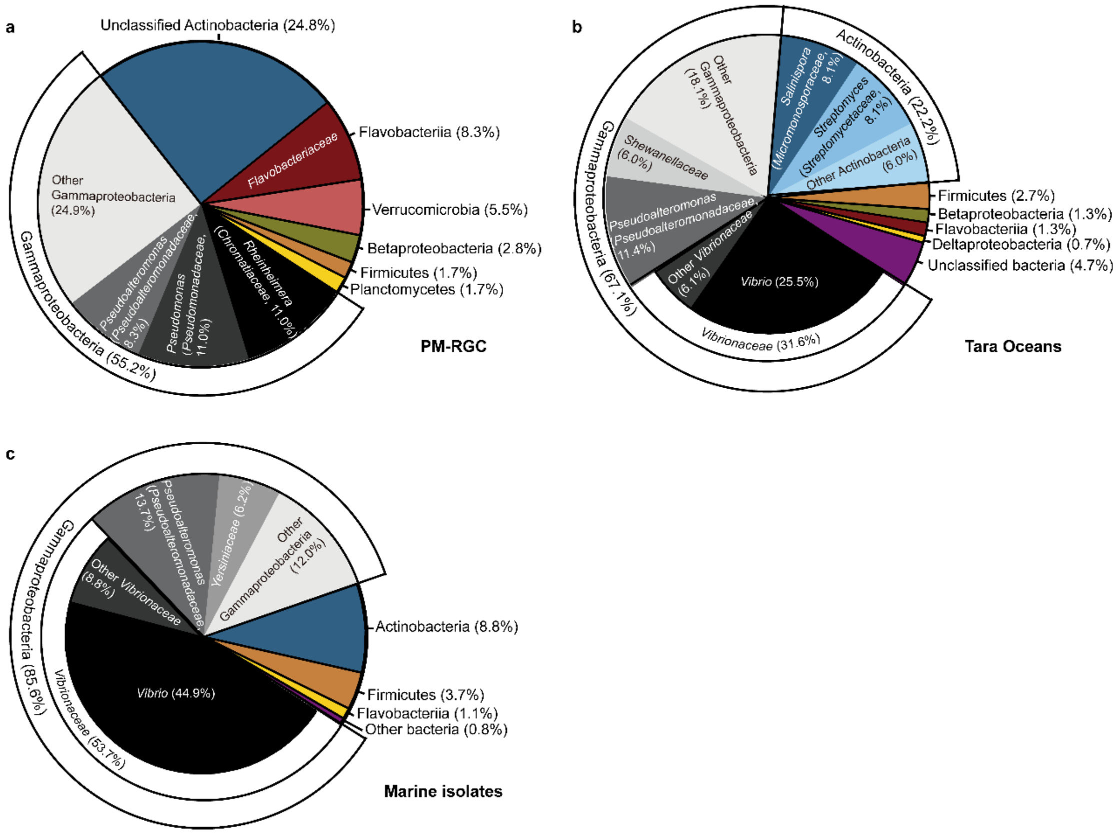

Supplementary Fig. 1 Distribution of AA10 LPMOs in marine bacteria. Percentage of distribution of AA10 LPMO-like sequences within each phylogenetic group in Polar marine reference gene catalog (PM-RGC) (**a**), Tara Oceans datasets (**b**) and marine bacterial isolates (**c**) is shown. AA10-like sequences were retrieved by BLASTP using the AA10 LPMOs from the marine strain ACAM 620 and the terrestrial *Serratia marcescens*[1] as queries (E-value < 10^{-5}, identity > 20% and coverage > 50%). BLAST searches against environmental metagenomes and marine isolates revealed that AA10 LPMOs are widespread among diverse bacterial phyla including Gammaproteobacteria and Actinobacteria. Bacteria harboring LPMO account for 1.6% and 1.7% of the total bacterial abundance in the PM-RGC and Tara Oceans metagenomes, respectively. Moreover, AA10 LPMOs are frequently found in marine bacteria belonging to genera *Vibrio* and *Pseudoalteromonas* of Gammaproteobacteria.

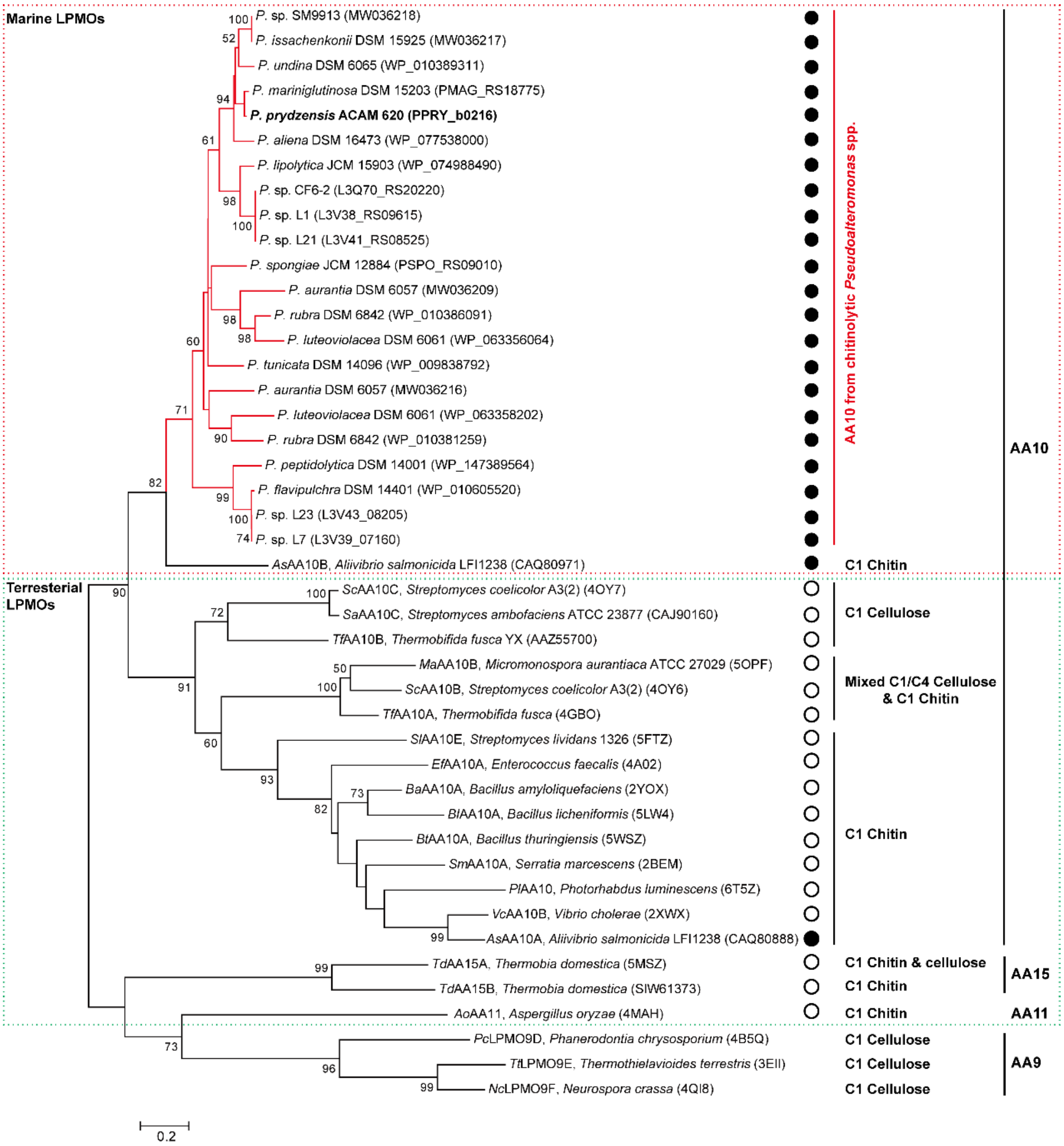

Supplementary Fig. 2 Phylogenetic analysis of LPMOs (predicted catalytic domains only) from chitinolytic *Pseudoalteromonas* spp. and characterized chitin-active LPMOs. The tree was constructed by the neighbor-joining method with a JTT-matrix-based model using 90 amino acid positions. Bootstrap analysis of 1,000 replicates is conducted and values above 50% are shown. The dominating substrate specificity and oxidative regioselectivity (C1, C4 or mixed C1/C4) for each cluster of characterized LPMOs are indicated. LPMOs from marine and terrestrial organisms are marked by solid and empty circles, respectively. All chitinolytic *Pseudoalteromonas* spp. contain at least one AA10 LPMO from the *cdc* cluster. Phylogenetic analysis suggested that marine LPMOs including the ones from *Pseudoalteromonas* spp. and the only characterized one from the marine bacterium *Aliivibrio salmonicida* LFI1238[2] are clustered within the AA10 family but form a separate group from characterized terrestrial AA10 homologs. Sequence identities shared by the LPMOs from chitinolytic *Pseudoalteromonas* spp. and characterized terrestrial AA10 LPMOs range from 20% to 34%.

Locus_tag	Annotation	Predicted cellular localization	CAZY
●PPRY_b0217	chitinase ChiA	Extracellular (SPI)	GH18, CBM5
PPRY_a0779	carbohydrate-binding protein	Extracellular (SPI)	CBM5
●PPRY_b0215	chitinase ChiC	Extracellular (SPI)	GH18, CBM5
●PPRY_b0216	LPMO	Extracellular (SPI)	AA10, CBM5
PPRY_a2075	DNA-binding protein	Periplasmic (SPI)	
PPRY_a2619	hypothetical protein	Extracellular (SPII)	
PPRY_a3391	TonB-dependent receptor	Outer Membrane (SPI)	
○PPRY_a3246	*N*-acetyl-β-hexosaminidase	Outer Membrane (SPI)	GH20
PPRY_a4305	hypothetical protein	Unknown	
PPRY_a3378	TonB-dependent receptor	Outer Membrane (SPI)	
PPRY_a0208	copper resistance protein CopC	Periplasmic (SPI)	
PPRY_a2158	flagellin	Extracellular	
PPRY_a3210	pilus assembly protein TapA	Extracellular	
PPRY_b0210	azurin	Periplasmic (SPI)	
PPRY_a1346	protein YceI	Extracellular (SPI)	
PPRY_a2156	flagellin	Extracellular	
PPRY_a0856	pectate lyase	Extracellular (SPI)	PL1, CBM13
PPRY_b0524	arylsulfatase	Periplasmic (SPI)	
PPRY_b0740	integrins alpha chain	Outer Membrane (SPI)	
PPRY_b0713	superoxide dismutase	Periplasmic	
PPRY_a2440	serine protease	Extracellular (SPI)	
PPRY_a2806	membrane protein	Outer Membrane (SPI)	
PPRY_a3980	flagellar motor protein MotA	Unknown (SPI)	
PPRY_a1396	outer membrane lipoprotein-sorting protein	Unknown (SPI)	
PPRY_a3851	alpha-amylase	Extracellular (SPI)	GH13
PPRY_a3254	adhesin	Extracellular (SPI)	
PPRY_a2910	pre-peptidase	Extracellular (SPI)	
PPRY_a3978	DUF3450 domain-containing protein	Outer Membrane	
PPRY_a2168	flagellar hook protein FlgE	Extracellular	
PPRY_a1919	acyl carrier protein	Cytoplasmic	
PPRY_a0020	50S ribosomal protein L7/L12	Unknown	
PPRY_a2747	cytochrome C	Periplasmic	
PPRY_b0790	TonB-dependent receptor	Outer Membrane (SPI)	
PPRY_a1745	peptidoglycan-associated lipoprotein	Outer Membrane (SPII)	
PPRY_b0111	hemin receptor	OuterMembrane (SPI)	
PPRY_b0750	hypothetical protein	Unknown (SPI)	
PPRY_a1432	flavodoxin	Cytoplasmic	
PPRY_b0749	hypothetical protein	Extracellular (SPI)	
PPRY_a1924	50S ribosomal protein L32	Cytoplasmic	
PPRY_a2397	DNA degradation protein EddB	Extracellular (SPI)	
PPRY_a2859	50S ribosomal protein L2	Cytoplasmic	
PPRY_a4418	translation elongation factor Tu	Cytoplasmic	
PPRY_a3255	hypothetical protein	Extracellular (SPII)	
PPRY_a1424	dihydrolipoamide succinyltransferase	Cytoplasmic	
PPRY_a2610	50S ribosomal protein L15	Cytoplasmic	
PPRY_a2186	T1SS-143 repeat domain-containing protein	Unknown	
PPRY_a2070	peroxidase	Cytoplasmic	
PPRY_a2807	membrane protein	Outer Membrane (SPI)	
PPRY_a4335	disulfide bond formation protein	Periplasmic (SPI)	
PPRY_a2320	Nikel transport family protein NikM	Unknown (SPI)	
PPRY_a3958	hypothetical protein	Unknown	
PPRY_a1360	lipase	Outer Membrane (SPII)	
PPRY_b0266	nucleoside diphosphate kinase	Cytoplasmic	
PPRY_a0918	hypothetical protein	Unknown (SPI)	
PPRY_a2862	50S ribosomal protein L3	Cytoplasmic	
PPRY_a0452	porin	Outer Membrane (SPI)	
PPRY_a2210	TonB-dependent siderophore receptor	Outer Membrane (SPI)	
PPRY_b0557	peptidase family S8A	Extracellular (SPI)	
PPRY_a2891	chemotaxis protein	Unknown	
PPRY_a0211	copper resistance protein CopB	Outer Membrane (SPI)	
PPRY_a4101	cysteine synthase A	Cytoplasmic	
PPRY_a2617	50S ribosomal protein L17	Cytoplasmic	
PPRY_a3976	cold-shock protein	Cytoplasmic	
PPRY_a1431	transcriptional repressor	Cytoplasmic	
PPRY_a2882	hypothetical protein	Outer Membrane (SPII)	
PPRY_a4336	disulfide bond formation protein	Periplasmic (SPI)	
PPRY_a2741	peptidase	Extracellular (SPII)	
PPRY_a2829	type I glutamate--ammonia ligase	Cytoplasmic	
PPRY_a0968	50S ribosomal protein L19	Cytoplasmic	
PPRY_a3447	pectate trisaccharide-lyase	Extracellular (SPI)	PL1, CBM13

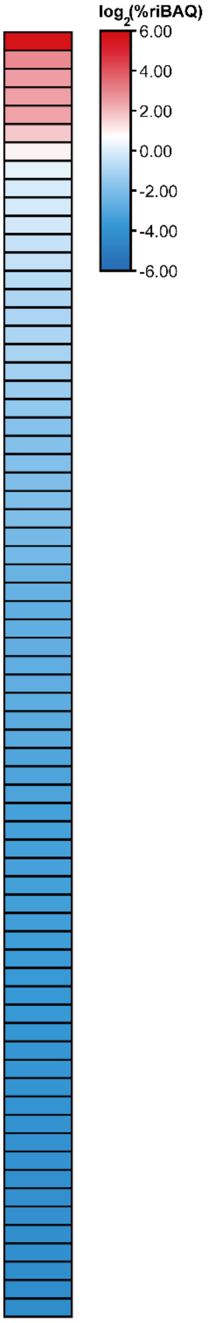

Supplementary Fig. 3 Heat map for identified proteins with relative abundance of more than 0.05% in the secretome of *P. prydzensis* ACAM 620 grown on colloidal chitin as the sole carbon source. The colors in the heat map indicate relative protein abundance, ranging from high (red) to low abundance (blue). The data are presented as $\log_2$ transformation of the mean values of two biological replicates for each protein. Seventy proteins showed relative abundance of more than 0.05% in the secretome of strain ACAM 620, accounting for 95.94% of the total protein abundance. The locus tag, protein annotation, CAZy family (glycosyl hydrolase (GH), carbohydrate-binding module (CBM), auxiliary activity (AA) and polysaccharide lyase (PL)) and predicted cellular localization are shown. Chitinolytic enzymes encoded by the *cdc* cluster are marked by solid circles and the other one by an empty circle. SpI, signal peptidase I cleavage site; SpII, signal peptidase II cleavage site.

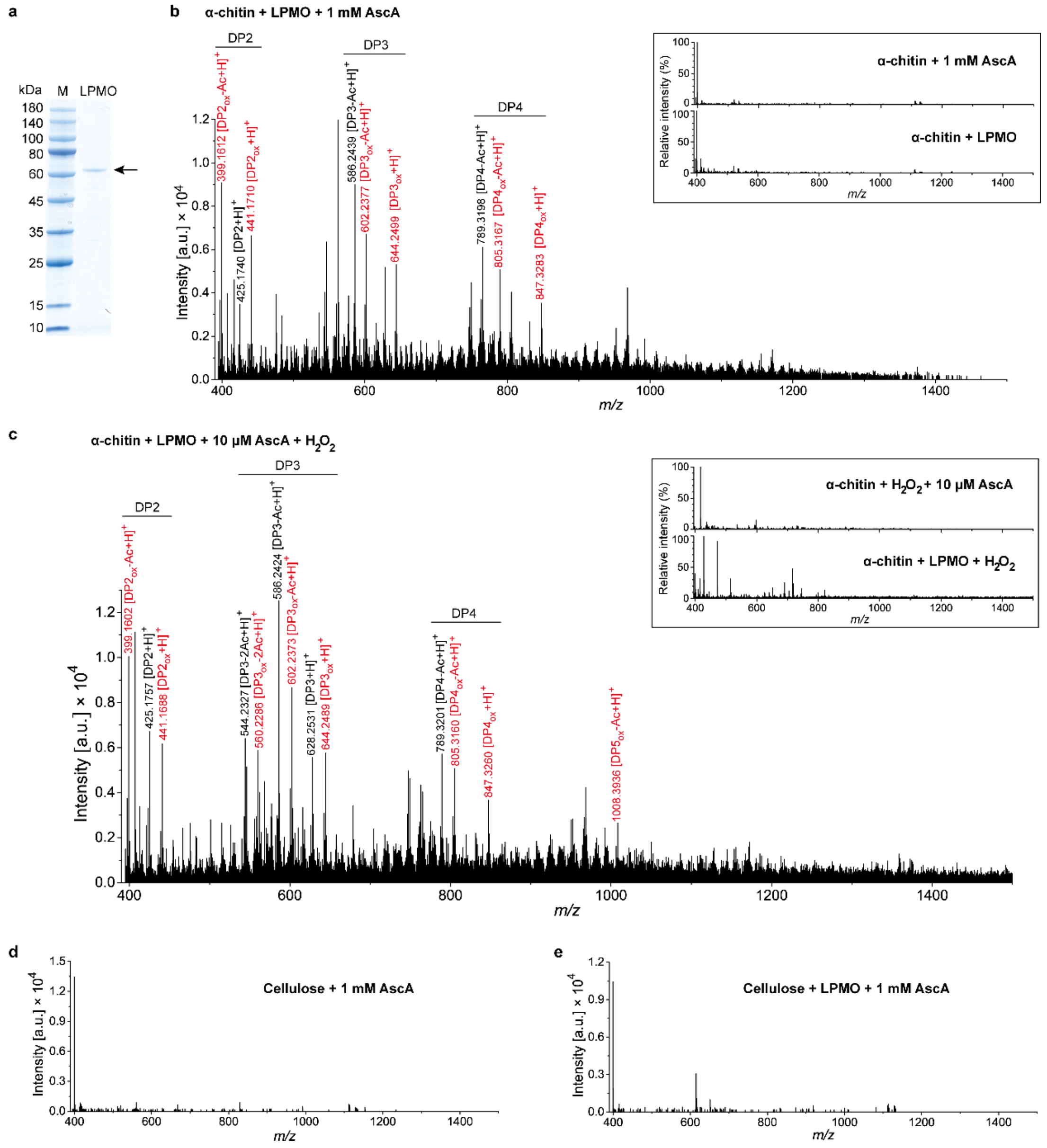

Supplementary Fig. 4 Expression and enzymatic activity analysis of the AA10 LPMO from *P. prydzensis* ACAM 620. a, SDS-PAGE analysis of purified recombinant LPMO (marked by an arrow) from *P. prydzensis* ACAM 620. The data shown are representatives of triplicate experiments. **b,** Positive-mode Q-TOF-MS spectrum of products generated by the LPMO acting on 0.2% (w/v) shrimp shell α-chitin in the presence of 1 mM AscA. Oxidized and non-oxidized chitooligosaccharides are labelled in red and black, respectively. The insets show the negative control reactions without AscA or LPMO, which did not generate detectable amounts of oxidized chitooligosaccharides. 100% relative intensity in the inserts represents 1.3 x 10^4 (control reaction without LPMO) and 1.4 x 10^4 (control reaction without AscA) a.u., respectively. **c,** Positive-mode Q-TOF-MS spectrum of products generated by the LPMO acting on 0.2% (w/v) shrimp shell α-chitin in the presence of 10 μM AscA and 100 μM H_2O_2. Oxidized and non-oxidized chitooligosaccharides are labelled in red and black, respectively. The insets show the negative control reactions without LPMO or AscA, which did not generate detectable amounts of oxidized chitooligosaccharides.

100% relative intensity in the inserts represents 2.3 x 10^4 (control reaction without LPMO) and 7.9 x 10^3 (control reaction without AscA) a.u., respectively. **d,** Positive-mode Q-TOF-MS spectrum of products obtained from the negative control reaction with 0.2% (w/v) cellulose and 1 mM AscA. No oxidized cello-oligosaccharides was detectable in the negative control reaction. **e,** Positive-mode Q-TOF-MS spectrum of products generated by the LPMO acting on 0.2% (w/v) cellulose in the presence of 1 mM AscA. No oxidized cello-oligosaccharides was detectable, suggesting that the LPMO has no cellulose-degrading activity. In **b-e**, the graphs show a representative MS spectrum of at least three independent replicates. See Supplementary Table 2 for a list of relevant products and their theoretical masses. Source data are provided as a Source Data file.

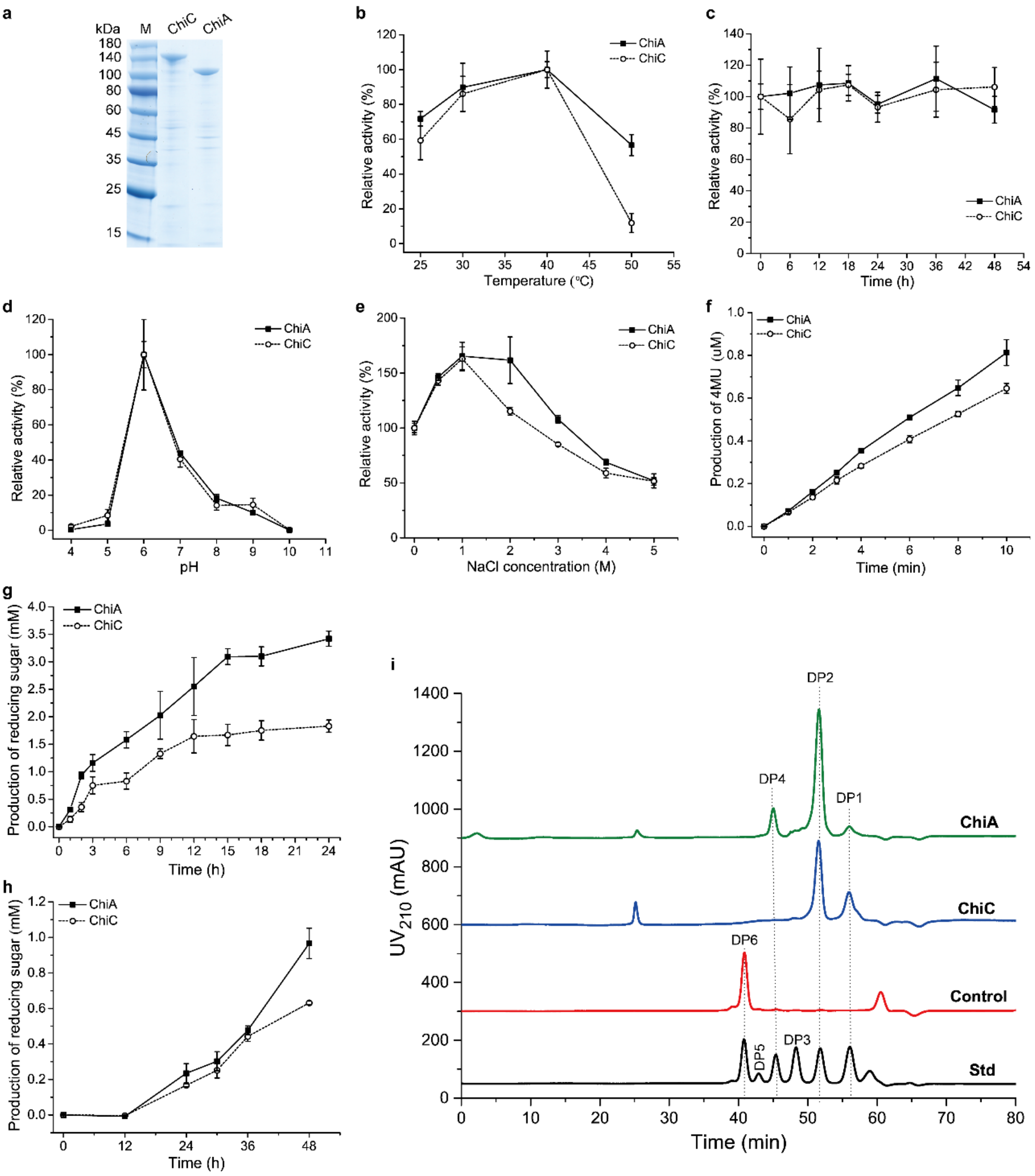

Supplementary Fig. 5 Functional analyses of recombinant chitinases, ChiC and ChiA, from *P. prydzensis* ACAM 620. a, SDS-PAGE analyses of purified recombinant enzymes, ChiC and ChiA. The theoretical molecular weights of ChiA and ChiC without signal peptides are 90.3 kDa and 110.6 kDa, respectively. The data shown are representatives of triplicate experiments. **b,** Effect of temperature on the activities of ChiA and ChiC against colloidal chitin. Both ChiA and ChiC showed the highest activity at 40°C and retained more than 59% of their highest activities at 25°C (the optimum growth temperature of strain ACAM 620). **c,** Effect of temperature on the stabilities of ChiA and ChiC. Both enzymes were incubated at 25°C for different time intervals, and their residual activities were measured at pH 6.0 and 25°C. Both ChiA and ChiC are stable at 25°C, retaining more than 90% activities after 48-h incubation at 25°C. **d,** Effect of pH on the activities of ChiA and ChiC. Both ChiA and ChiC showed the highest activity at pH 6.0 and retained less than 20% of their highest

activities at pH 8.0 (near the pH of seawater). **e,** Effect of NaCl on the activities of ChiA and ChiC. The activities of both enzymes could be stimulated by 0.5 M NaCl by approximately 1.4-fold, consistent with their marine origin. **f,** Generation of 4-methylumbelliferone (4MU) from 4MU-(GlcNAc)$_2$ by ChiA and ChiC over time. Hydrolysis of 4MU-(GlcNAc)$_2$ was carried out at 25°C and pH 6.0, and the concentrations of the enzyme and substrate used were 0.1 μM and 0.1 mM, respectively. **g,** Generation of reducing sugars from colloidal chitin by ChiA and ChiC over time. Reactions were carried out at 25°C by adding 5 μM ChiA or ChiC to a reaction solution containing 10 mg/mL colloidal chitin and 0.5 M NaCl in 10 mM Tris-HCl (pH 6.0). **h,** Generation of reducing sugars from crystalline α-chitin by ChiA and ChiC. Reactions were carried out at 25°C by adding 10 μM ChiA or ChiC to a reaction solution containing 6 mg/mL α-chitin and 0.5 M NaCl in 10 mM Tris-HCl (pH 6.0). Data shown in **b-h** are mean ± SD (n = 3 independent experiments). **i,** Analysis of the hydrolysis products from (GlcNAc)$_6$ by ChiA and ChiC. Hydrolysis of (GlcNAc)$_6$ was carried out at 25°C and pH 6.0 for 6 h and the concentrations of the enzyme and substrate used were 1 μM and 2.5 mg/ml, respectively. DP, degree of polymerization; Std, standard mix of (GlcNAc)$_{1\text{-}6}$. The products from (GlcNAc)$_6$ degradation by ChiA are almost even-number chitooligomers, (GlcNAc)$_2$ and (GlcNAc)$_4$, suggesting that ChiA functions as an exochitinase. The products from (GlcNAc)$_6$ degradation by ChiC are GlcNAc and (GlcNAc)$_2$, suggesting that ChiC functions as an endochitinase. The graph shows representative data of at least three independent replicates. Source data are provided as a Source Data file.

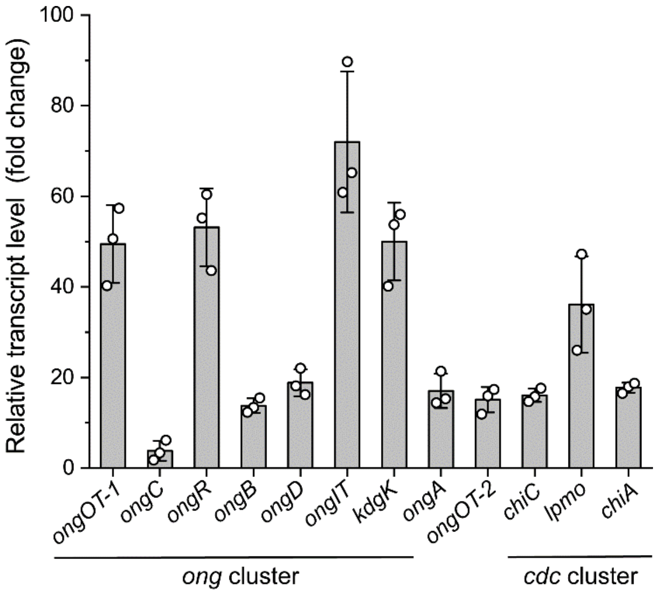

Supplementary Fig. 6 RT-qPCR assay of the transcriptions of *ongOT-2* and genes from the *ong* cluster and the *cdc* cluster of strain ACAM 620 in the minimal medium supplemented with 0.5% (w/v) colloidal chitin. The bacterium cultivated in the minimal medium supplemented with glucose was used as the control. The *rpoD* gene was used as an internal reference. Data are presented as mean ± SD (n = 3 independent experiments). Source data are provided as a Source Data file.

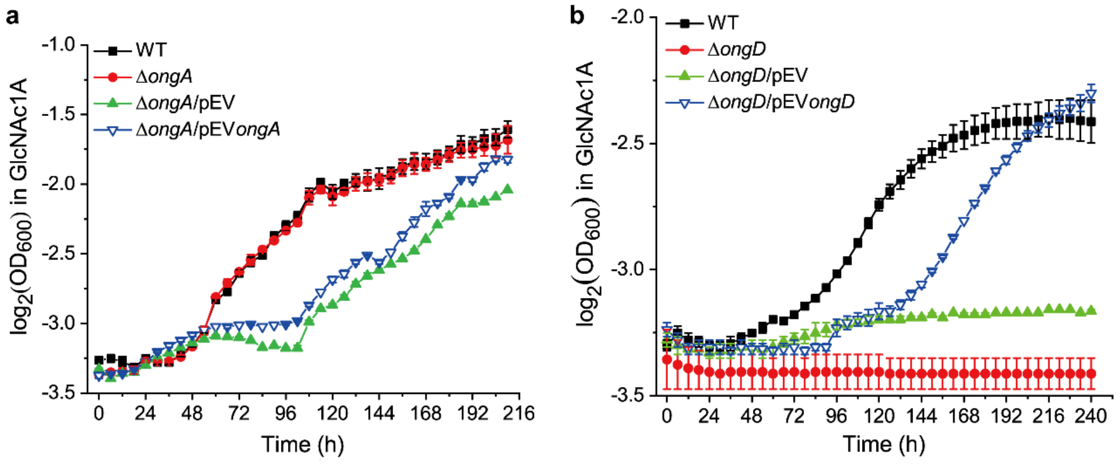

Supplementary Fig. 7 Growth phenotype analysis of the *ongA*-deletion (a) and the *ongD*-deletion (b) mutants of strain ACAM 620. Wild-type strain (WT), mutant strains and complemented strains of mutants were all grown at 25°C in the minimal medium supplemented with 10 mM GlcNAc1A. Deletion mutant strains with the empty plasmid pEV were used a control. The *y*-axes in **a** and **b** represent $\log_2$ transformation of the OD_{600} value. Data shown in **a** and **b** are mean ± SD (n = 2 independent experiments). Source data are provided as a Source Data file.

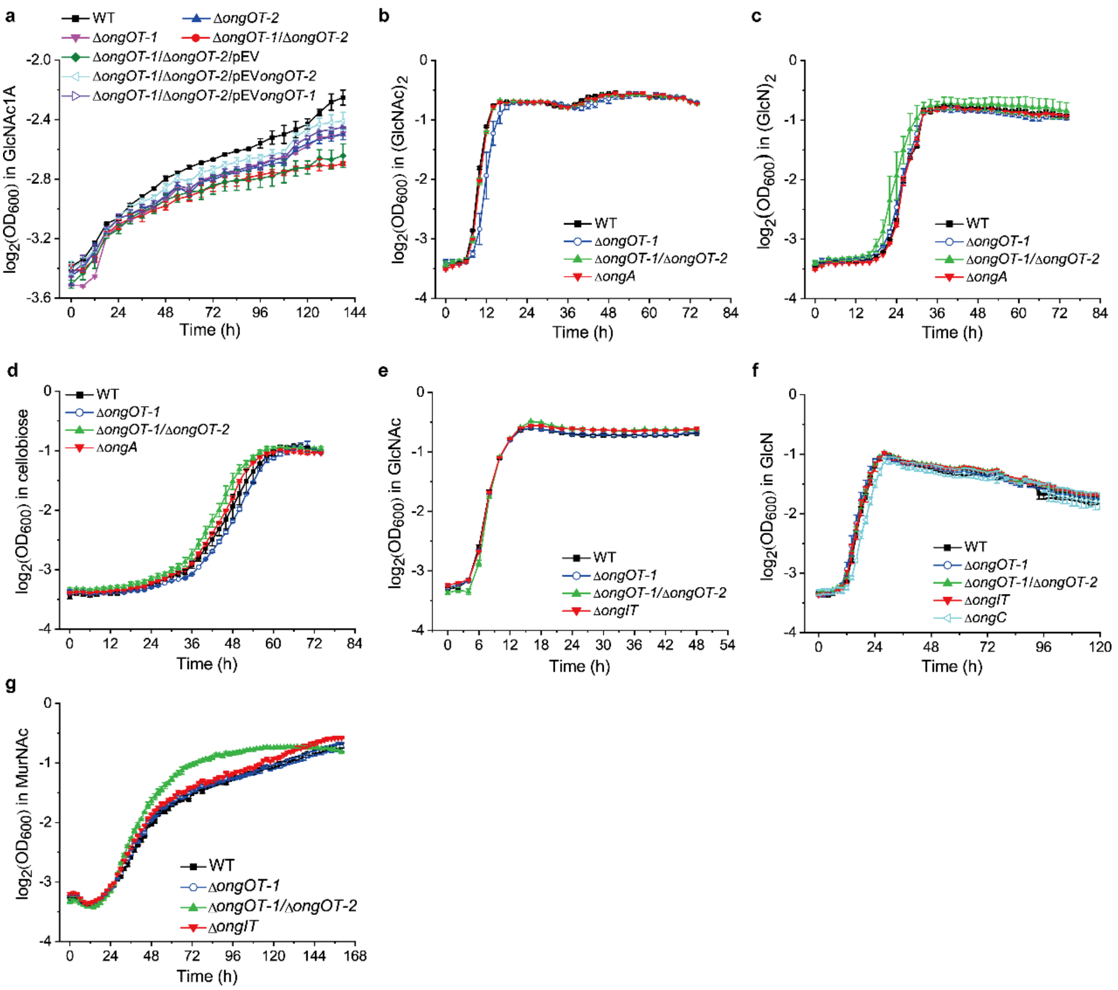

Supplementary Fig. 8 Growth phenotype analysis of the transporter-deletion mutants of strain ACAM 620. Growth of the wild-type strain (WT) and its mutant strains including △*ongOT-1*, △*ongOT-1*/△*ongOT-2* and △*ongIT* (and/or △*ongOT-2* and/or △*ongA* and/or △*ongC*) at 25°C in the minimal medium supplemented with GlcNAc1A (**a**), $(GlcNAc)_2$ (**b**), $(GlcN)_2$ (**c**), cellobiose (**d**), GlcNAc (**e**), D-glucosamine (GlcN) (**f**) or *N*-acetylmuramic acid (MurNAc) (**g**) at a concentration of 10 mM as the sole carbon source. Among various carbon sources tested, strain ACAM 620 showed no detectable growth on sodium carboxymethyl cellulose, *N*-acetylneuraminic acid (Neu5Ac), *N*-acetyl-D-mannosamine (ManNAc), *N*-acetyl-D-glutamate, *N*-acetyl-D-serine, *N*-acetyl-D-methionine, D-gluconate, D-mannosamine, D-galactosamine, D-serine, D-threonine or D-arginine at a concentration of 10 mM as a sole carbon source. The *y*-axes in **a-g** represent log_2 transformation of the OD_{600} value. Data shown in **a-g** are mean ± SD (n = 2 independent experiments). Source data are provided as a Source Data file.

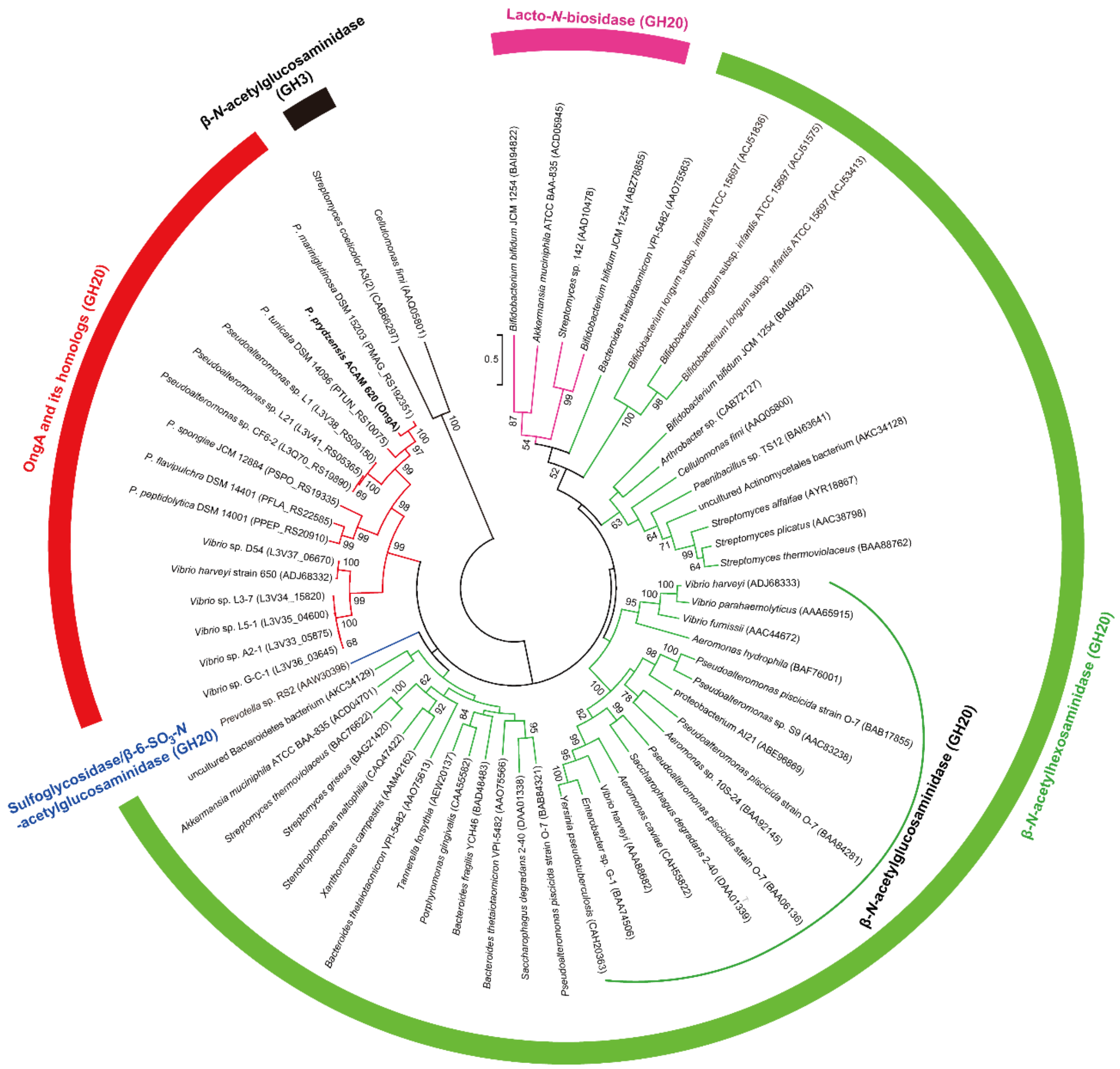

Supplementary Fig. 9 Phylogenetic analysis of OngA and its homologs and characterized GH20 glycoside hydrolases. The tree was constructed by the maximum-likelihood method with a WAG-based model using 264 amino acid positions. Bootstrap analysis of 1,000 replicates is conducted and values above 50% are shown. β-*N*-acetylglucosaminidases from the GH3 family were used as the outgroup. Except for the sequence (ADJ68332) from *Vibrio harveyi* strain 650 without determined genome sequence, all other homologs to OngA (colored in red) are from the *ong* clusters of their respective bacterial strains.

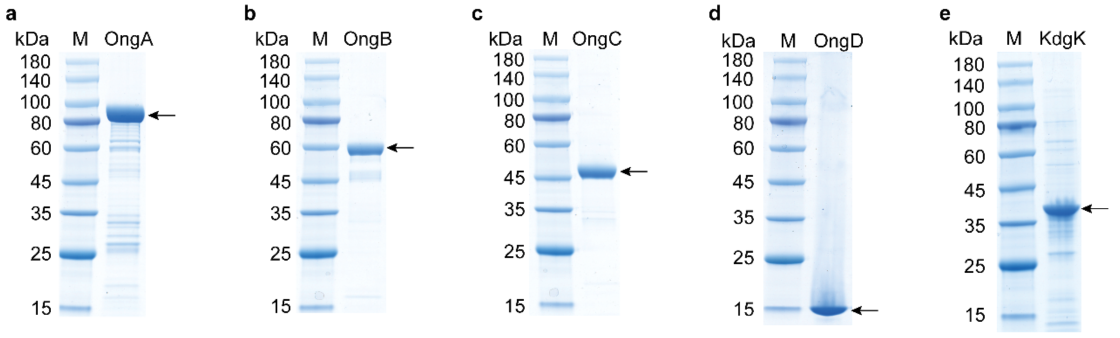

Supplementary Fig. 10 SDS-PAGE analyses of purified recombinant enzymes, OngA (a), OngB (b), OngC (c), OngD (d) and KdgK (e) from *P. prydzensis* ACAM 620. The theoretical molecular weights of these recombinant proteins are 84.2 kDa (OngA without a predicted signal peptide), 53.0 kDa (OngB), 45.4 kDa (OngC), 13.7 kDa (OngD) and 35.3 kDa (KdgK), respectively. The target proteins are indicated by arrows. Data shown in **a-e** are representatives of triplicate experiments. Source data are provided as a Source Data file.

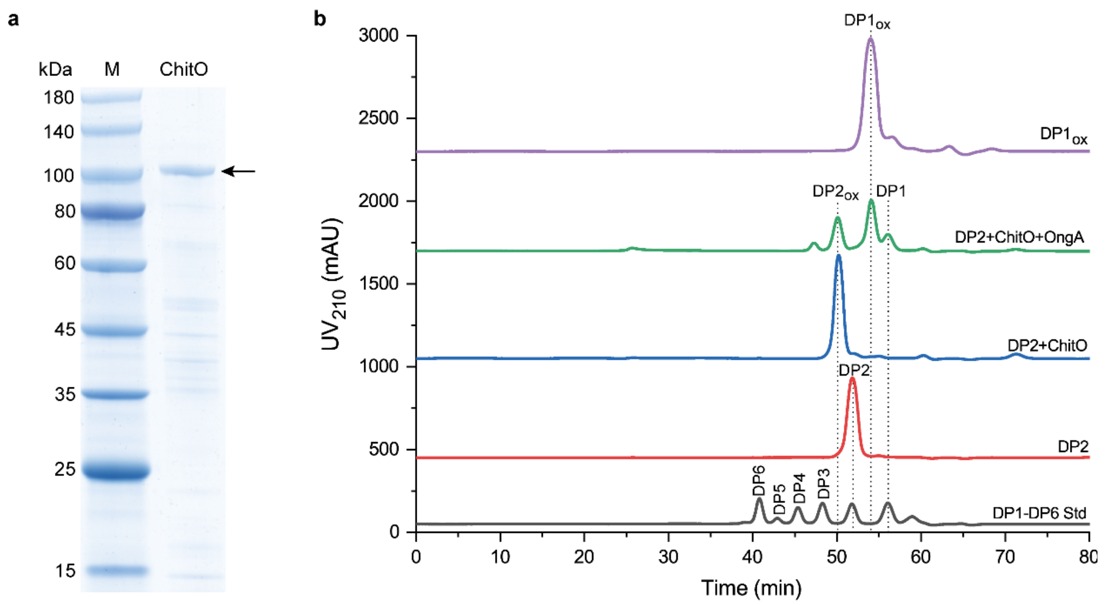

Supplementary Fig. 11 Analysis of the products generated by the recombinant OngA acting on GlcNAc-GlcNAc1A. a, SDS-PAGE analysis of the purified recombinant chitooligosaccharide oxidase, ChitO (marked by an arrow), from *Fusarium graminearum*[3] fused with an MBP tag. **b,** The preparation of GlcNAc-GlcNAc1A from $(GlcNAc)_2$ by ChitO and analysis of the products released from GlcNAc-GlcNAc1A by OngA. DP, degree of polymerization; $DP1_{ox}$, GlcNAc1A; $DP2_{ox}$, GlcNAc-GlcNAc1A. Data shown in **a** and **b** are representatives of triplicate experiments. Source data are provided as a Source Data file.

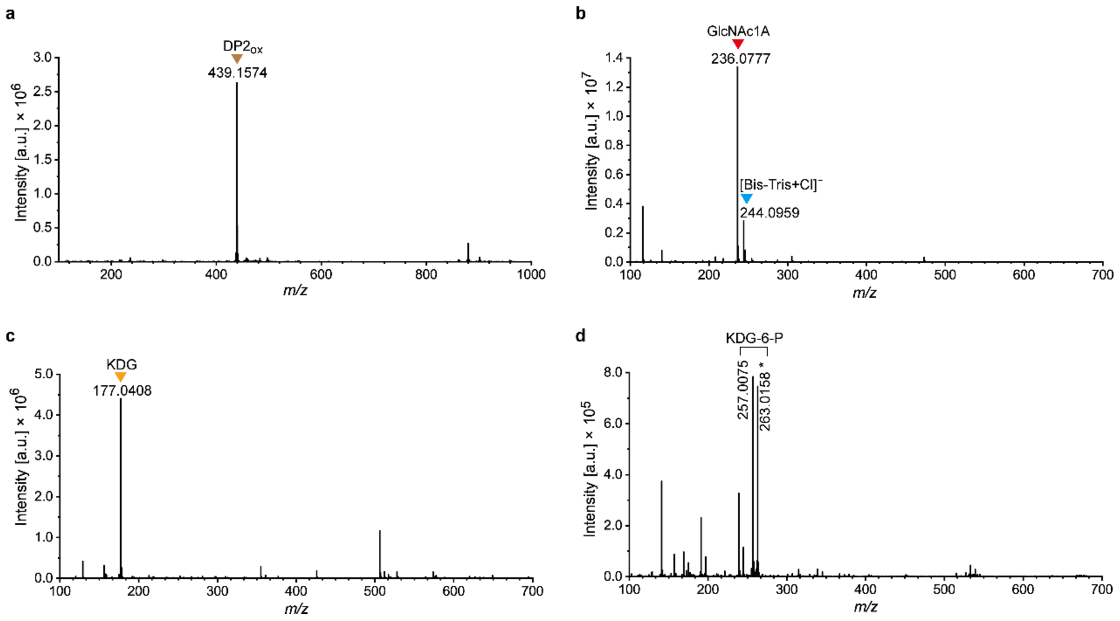

Supplementary Fig. 12 Negative-mode Q-TOF-MS spectra of GlcNAc-GlcNAc1A (a), GlcNAc1A (b), KDG (c) and KDG-6-P (d). GlcNAc-GlcNAc1A ($DP2_{ox}$) was prepared from chitobiose by using the chitooligosaccharide oxidase ChitO from *Fusarium graminearum*[3]. In **a-d**, all labeled MS peaks refer to $[M-H]^-$ ions in addition to the deprotonated lithium adducts of KDG-6-P (*m/z* 263.0158, marked by a star). The graphs show a representative MS spectrum of at least three independent replicates. See Supplementary Table 4 for a list of relevant products and their theoretical masses. Source data are provided as a Source Data file.

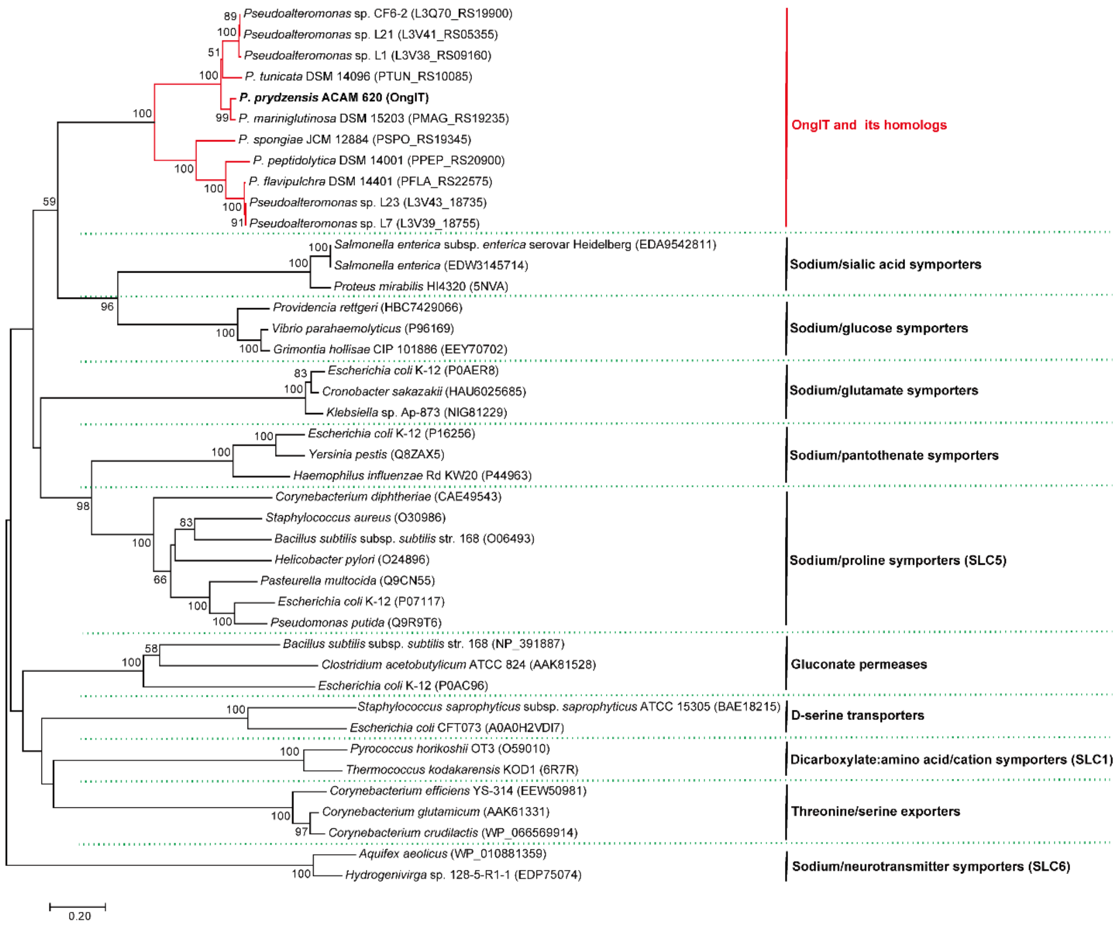

Supplementary Fig. 13 Phylogenetic analysis of OngIT and its homologs and characterized prokaryotic amino acid and sugar transporters. The tree was constructed by the neighbor-joining method with a Poisson model using 240 amino acid positions. Bootstrap analysis of 1,000 replicates is conducted and values above 50% are shown. All homologs to OngIT (colored in red) are from the *ong* clusters of their respective bacterial strains.

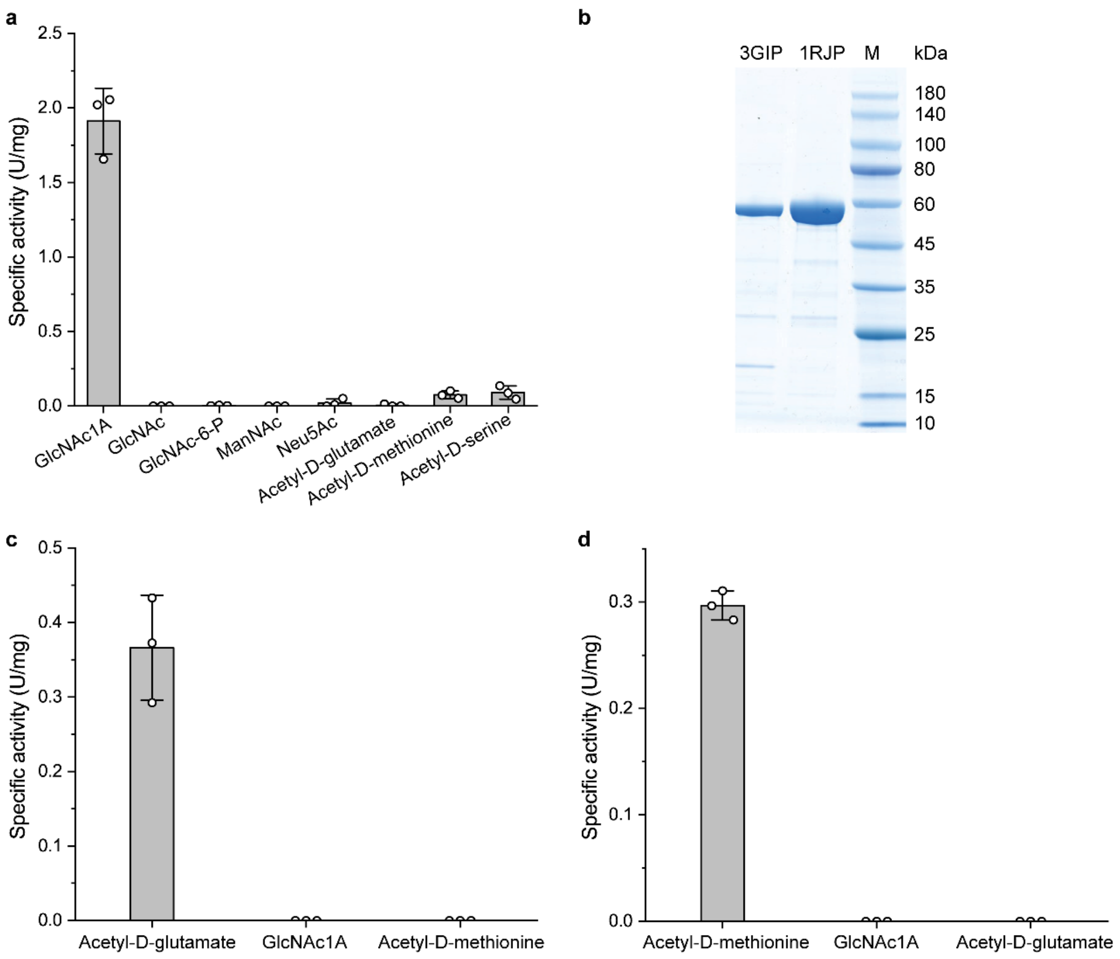

Supplementary Fig. 14 Substrate specificity analyses of OngB and two D-aminoacylases from the *N*-acetyl-D-glutamate deacetylase family. a, Substrate specificity analysis of OngB. ManNAc, *N*-acetyl-D-mannosamine; Neu5Ac, *N*-acetylneuraminic acid. Reactions were carried out at pH 7.5 and 25°C for 30 min and the concentrations of the enzyme and substrate used were 5 µM and 25 mM, respectively. **b,** SDS-PAGE analyses of purified recombinant D-aminoacylases from *Bordetella bronchiseptica* (PDB code 3GIP)[4] and *Alcaligenes faecalis* (PDB code 1RJP)[5], respectively. The data shown are representatives of triplicate experiments. **c,** Substrate specificity analysis of the D-aminoacylase from *B. bronchiseptica*. *B. bronchiseptica* D-aminoacylase could deacetylate *N*-acetyl-D-glutamate but had no activity towards GlcNAc1A. Reactions were carried out at pH 7.5 and 30°C for 20 h and the concentrations of the enzyme and substrate used were 1 µM and 25 mM, respectively. **d,** Substrate specificity analysis of the D-aminoacylase from *A. faecalis*. *A. faecalis* D-aminoacylase could deacetylate *N*-acetyl-D-methionine but had no activity towards GlcNAc1A. Reactions were carried out at pH 7.5 and 30°C for 20 h and the concentrations of the enzyme and substrate used were 1 µM and 25 mM, respectively. Data shown in **a**, **c** and **d** are mean ± SD (n = 3 independent experiments). Source data are provided as a Source Data file.

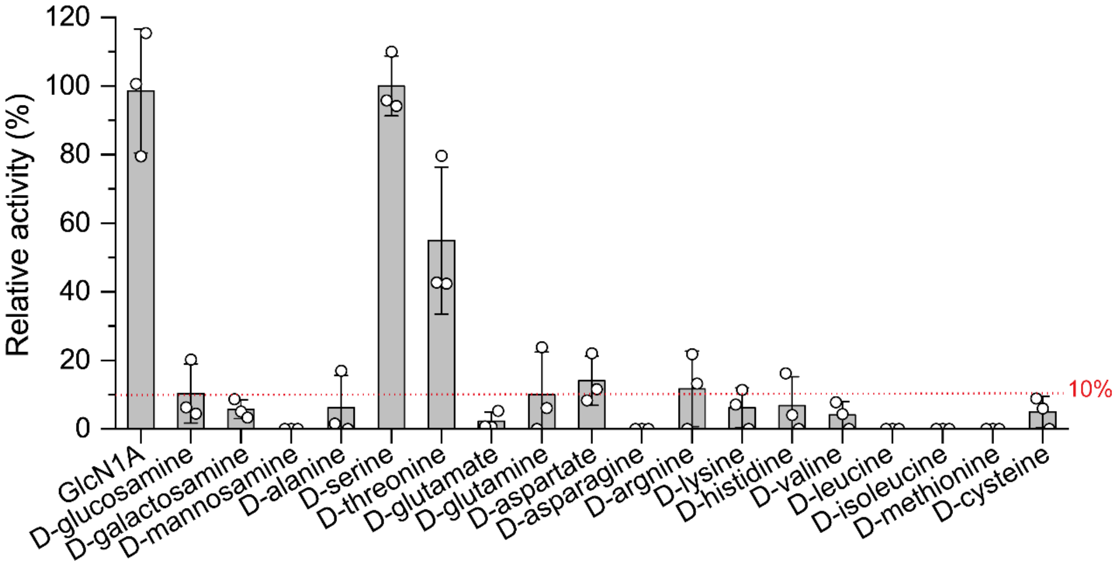

Supplementary Fig. 15 Substrate specificity analysis of OngC. OngC could also deaminate D-serine and D-threonine in addition to GlcN1A. The specific activity of OngC against GlcN1A (1.41 ± 0.21 U/mg) was defined as 100%. Reactions were carried out at pH 7.5 and 25°C for 30 min and the concentrations of the enzyme and substrate used were 0.5 μM and 10 mM, respectively. Data are presented as mean ± SD (n = 3 independent experiments). Source data are provided as a Source Data file.

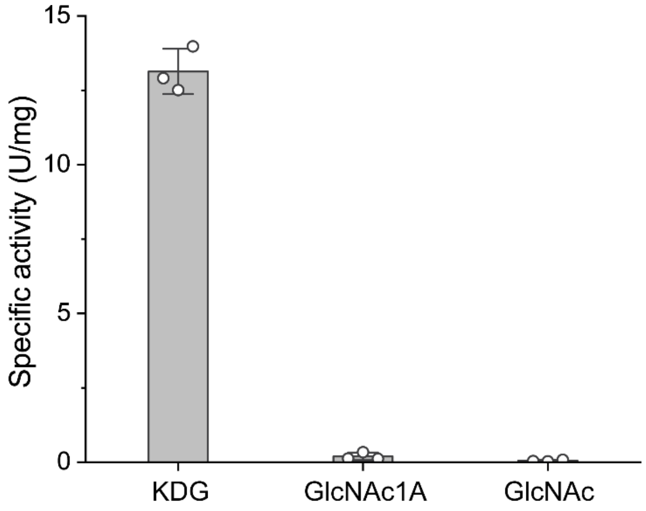

Supplementary Fig. 16 Substrate specificity analysis of KdgK. Reactions were carried out at pH 7.5 and 25°C for 5 min and the concentrations of the enzyme and substrate used were 0.05 μM and 1 mM, respectively. Data are presented as mean ± SD (n = 3 independent experiments). Source data are provided as a Source Data file.

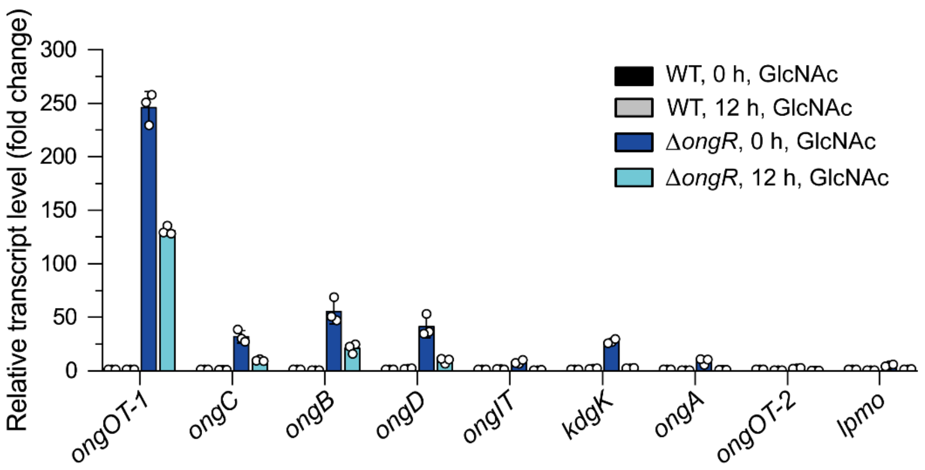

Supplementary Fig. 17 RT-qPCR assay of the transcriptions of *lpmo*, *ongOT-2* and genes from the *ong* cluster in the WT and Δ*ongR* mutant strains in response to 0.2% (w/v) GlcNAc in the minimal medium. Values are expressed as fold change compared to pre-cultures of the WT strain in the minimal medium supplemented with 0.2% (w/v) glucose. The *rpoD* gene was used as an internal reference. Data are presented as mean ± SD (n = 3 independent experiments). Source data are provided as a Source Data file.

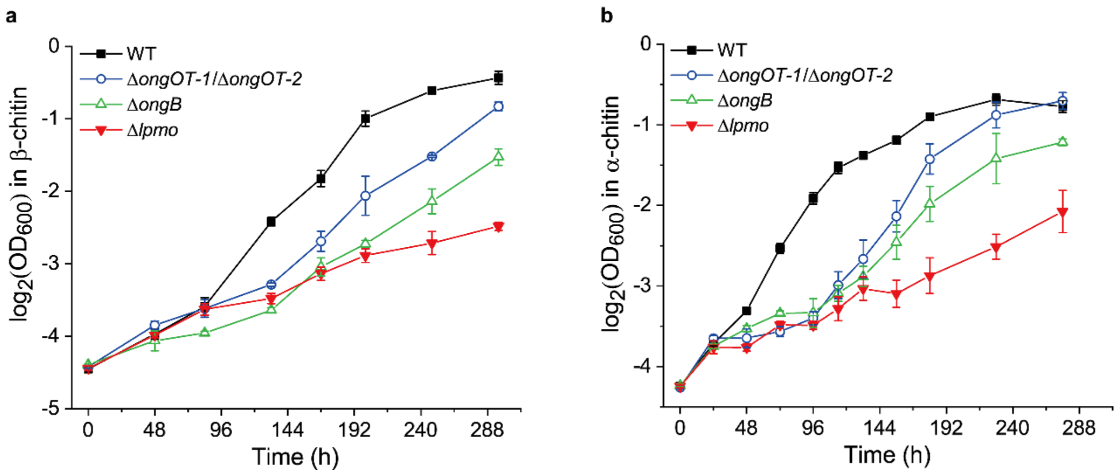

Supplementary Fig. 18 Growth curves of the *lpmo*-deletion, the *ongB*-deletion and the *ongOT-1/ongOT-2* double deletion mutants of strain ACAM 620 on β-chitin flakes (a) or α-chitin powder (b) as the sole carbon source. Wild-type strain (WT) and its mutants were grown at 25°C in the minimal medium supplemented with 0.2% (w/v) β-chitin flakes or 0.2% (w/v) α-chitin powder. The *y*-axes in **a** and **b** represent $\log_2$ transformation of the OD_{600} value. Data shown in **a** and **b** are mean ± SD (n = 3 independent experiments). Source data are provided as a Source Data file.

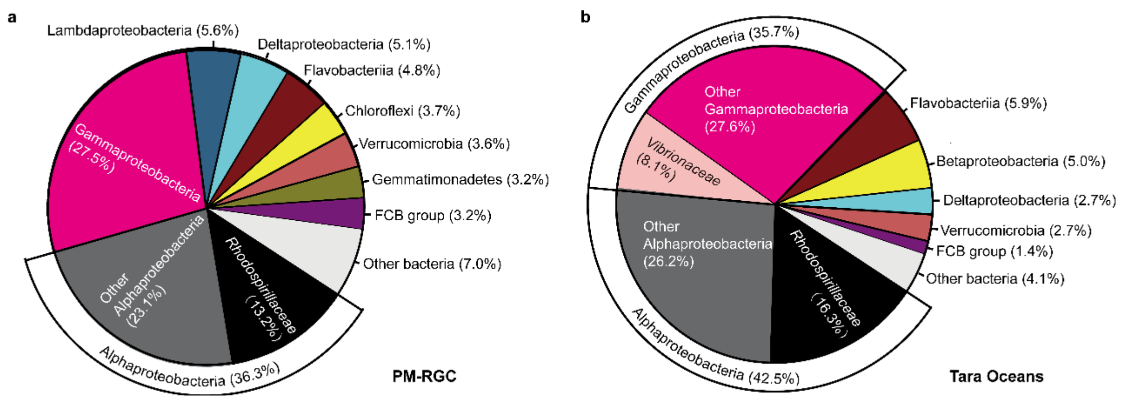

Supplementary Fig. 19 Distribution of OngB-like sequences in marine bacteria. Percentage of distribution of OngB-like sequences within each phylogenetic group in PM-RGC (**a**) and Tara Oceans datasets (**b**) is shown. OngB-like sequences were retrieved by BLASTP using the OngB from the marine strain ACAM 620 as the query (E-value < 10^{-5} and identity > 45%). BLAST searches against environmental metagenomes revealed that OngB-like sequences are widespread among diverse bacterial phyla including Alphaproteobacteria and Gammaproteobacteria, accounting for 7.0% and 2.6% of the total bacterial abundance in the PM-RGC and Tara Oceans metagenomes, respectively.

Supplementary Tables

Supplementary Table 1 The utilization of different carbon sources by marine *Pseudoalteromonas* spp. as the sole carbon source.

Strain[a]	Isolation source	The closest relative (16S identity)	Carbon source[b]					Gene/gene cluster[c]	
			D-gluconate	β-chitin	Colloidal chitin	GlcNAc	GlcNAc1A	*lpmo*	*ong* cluster
P. prydzensis ACAM 620	Sea ice from the Antarctic		ND	0.92 ± 0.02	0.90 ± 0.03	0.59 ± 0.01	0.26 ± 0.01	+	+
P. peptidolytica DSM 14001	Surface seawater from the Sea of Japan		ND	0.94 ± 0.01	0.69 ± 0.05	0.42 ± 0.03	0.12 ± 0.01	+	+
P. flavipulchra DSM 14401	Surface seawater off Nice, France		ND	1.46 ± 0.03	0.54 ± 0.07	0.32 ± 0.01	ND	+	+
P. sp. CF6-2	Deep-sea sediment from the South China Sea	*Pseudoalteromonas arabiensis* JCM 17292 (99.86%)	ND	1.00 ± 0.05	0.45 ± 0.01	0.64 ± 0.01	0.40 ± 0.02	+	+
P. sp. L1	Surface seawater from the Yellow Sea, China	*Pseudoalteromonas arabiensis* JCM 17292 (98.94%)	ND	0.82 ± 0.02	0.64 ± 0.01	0.66 ± 0.01	0.17 ± 0.01	+	+
P. sp. L7	Surface seawater from the Yellow Sea, China	*Pseudoalteromonas flavipulchra* NCIMB 2033 (99.23%)	ND	1.61 ± 0.03	0.71± 0.02	0.93 ± 0.09	0.24 ± 0.01	+	+
P. sp. L21	Surface seawater from the Yellow Sea, China	*Pseudoalteromonas arabiensis* JCM 17292 (99.17%)	ND	0.32 ± 0.02	0.59 ± 0.02	0.65 ± 0.02	0.31 ± 0.01	+	+
P. sp. L23	Surface seawater from the Yellow Sea, China	*Pseudoalteromonas piscicida* JCM 20779 (99.40%)	ND	1.61 ± 0.03	0.52 ± 0.01	0.74 ± 0.04	0.26 ± 0.01	+	+
P. aliena DSM 16473	Seawater from the Sea of Japan		ND	ND	0.25 ± 0.06	0.67 ± 0.08	ND	+	-
P. aurantia DSM 6057	Surface seawater off Nice, France		ND	ND	0.45 ± 0.04	0.64 ± 0.01	ND	+	-
P. issachenkonii DSM 15925	Brown alga in the Pacific Ocean		ND	ND	0.44 ± 0.03	0.35± 0.05	ND	+	-
P. lipolytica JCM 15903	The Yangtze River estuary near the East China Sea		ND	ND	0.63 ± 0.01	0.36 ± 0.03	ND	+	-
P. luteoviolacea DSM 6061	Surface seawater near Nice, France		ND	ND	0.30 ± 0.22	0.33 ± 0.15	ND	+	-
P. mariniglutinosa DSM 15203	Diatom from the Marseille Gulf		ND	0.11 ± 0.01 (0.57 ± 0.01)	0.24 ± 0.05	0.49 ± 0.01	0.28 ± 0.01	+	+
P. rubra DSM 6842	The Mediterranean seawater off Nice, France		ND	ND	0.19 ± 0.06	0.51 ± 0.10	ND	+	-
P. spongiae JCM 12884	Sponge in Hong Kong waters, China		ND	0.12 ± 0.01 (0.62 ± 0.01)	0.30 ± 0.01	0.48 ± 0.18	0.16 ± 0.01	+	+
P. tunicata DSM 14096	Tunicate from water off the western coast of Sweden		ND	ND	0.33 ± 0.02	0.25 ± 0.05	ND	+	+
P. undina DSM 6065	Seawater off the coast of Northern California, U.S.		ND	ND	0.54 ± 0.01	0.40 ± 0.13	ND	+	-
P. sp. SM9913	Deep-sea sediment from the Okinawa Trough	*Pseudoalteromonas atlantica* NBRC 103033 (99.79%)	ND	ND	0.26 ± 0.02	0.40 ± 0.11	ND	+	-

[a] All non-type strains of *Pseudoalteromonas* including strains CF6-2, L1, L7, L21, L23 and SM9913 were isolated by our laboratory, and all type strains of *Pseudoalteromonas* were purchased from DSMZ or JCM.

[b] Bacterial strains with no detectable growth on a given carbon source after 14-day cultivation at 25°C were labeled by "ND"; for bacterial strains grown on a given carbon source, the maximum OD_{600} values of bacterial cells on GlcNAc and GlcNAc1A at 25°C were shown, and the OD_{600} values of their 7-day cultures (and 14-day cultures in parentheses) on β-chitin as well as the OD_{600} values of 4-day cultures on colloidal chitin at 25°C were shown. All data shown are mean ± SD (n = 2 independent experiments). Source data are provided as a Source Data file.

[c] +, presence; -, absence.

Supplementary Table 2 Theoretical monoisotopic masses of various possible ions for oxidized and non-oxidized chitooligosaccharides generated by the LPMO from *P. prydzensis* ACAM 620 acting on chitin.

Product type	Product	Formula	$[M+H]^+$	$[M+Na]^+$	$[M\text{-}Ac+H]^+$	$[M\text{-}2Ac+H]^+$
Oxidized chitooligosaccharides	$DP2_{ox}$	$C_{16}H_{28}N_2O_{12}$	441.1715	463.1534	399.1609	357.1504
	$DP3_{ox}$	$C_{24}H_{41}N_3O_{17}$	644.2509	666.2328	602.2403	560.2297
	$DP4_{ox}$	$C_{32}H_{54}N_4O_{22}$	847.3302	869.3122	805.3197	763.3091
	$DP5_{ox}$	$C_{40}H_{67}N_5O_{27}$	1050.4096	1072.3916	1008.3991	966.3885
Chitooligosaccharides	DP2	$C_{16}H_{28}N_2O_{11}$	425.1766	447.1585	383.1660	341.1555
	DP3	$C_{24}H_{41}N_3O_{16}$	628.2560	650.2379	586.2454	544.2348
	DP4	$C_{32}H_{54}N_4O_{21}$	831.3353	853.3173	789.3248	747.3142
	DP5	$C_{40}H_{67}N_5O_{26}$	1034.4147	1056.3966	992.4041	950.3936

[a] M-Ac, oxidized and non-oxidized chitooligosaccharides lacking one acetyl group; M-2Ac, oxidized and non-oxidized chitooligosaccharides lacking two acetyl groups.

Supplementary Table 3 Key genes involved in oxidative degradation of chitin in *P. prydzensis* ACAM 620[a].

Gene cluster	Locus_tag	Gene	Length (aa)	Annotation	Signal peptide	Cellular localization	Expression	
							RNA (Log$_2$(GlcNAc1A/glucose))	Protein (%riBAQ in chitin)
cdc	PPRY_b0215	*chiC*	1080	GH18 chitinase	1-27 aa	Extracellular	-1.60 ± 0.48	5.75 ± 0.47
	PPRY_b0216	*lpmo*	531	AA10 LPMO	1-29 aa	Extracellular	--	5.59 ± 1.61
	PPRY_b0217	*chiA*	875	GH18 chitinase	1-21 aa	Extracellular	--	51.43 ± 2.33
ong	PPRY_b0286	*ongOT-1*	979	TonB-dependent receptor	1-34 aa	Outer membrane	8.13 ± 0.26	0.005 ± 0.002
	PPRY_b0288	*ongC*	415	Putative D-amino acid deaminase	--	Cytoplasmic	5.44 ± 0.43	--
	PPRY_b0289	*ongR*	283	MurR/RpiR family transcriptional regulator	--	Cytoplasmic	4.03 ± 0.12	--
	PPRY_b0290	*ongB*	489	Putative D-aminoacylase	--	Cytoplasmic	4.44 ± 0.16	--
	PPRY_b0291	*ongD*	127	RidA family protein	--	Cytoplasmic	4.85 ± 0.05	--
	PPRY_b0294	*ongIT*	482	Sodium:solute symporter family protein	--	Inner membrane	5.61 ± 1.4	--
	PPRY_b0295	*kdgK*	318	Sugar kinase	--	Unknown	5.80 ± 1.3	--
	PPRY_b0297	*ongA*	770	GH20 β-hexosaminidase	1-21 aa	Periplasmic	4.58 ± 0.67	--
Other genes	PPRY_b0790	*ongOT-2*	1004	TonB-dependent receptor	1-30 aa	Outer membrane	1.71 ± 0.46	0.154 ± 0.02
	PPRY_a1992	*kdgA-1*	206	Putative 2-dehydro-3-deoxy-phosphogluconate aldolase	--	Cytoplasmic	--	0.021 ± 0.005
	PPRY_b1070	*kdgA-2*	207	Putative 2-dehydro-3-deoxy-phosphogluconate aldolase	--	Cytoplasmic	--	--

[a] --, undetectable.

Supplementary Table 4 Theoretical monoisotopic masses of various possible ions for analytes in this study.

Compound	Formula	$[M-H]^-$	$[M+Cl]^-$	$[M-2H+Li]^-$
$DP2_{ox}$	$C_{16}H_{28}N_2O_{12}$	439.1570		
GlcNAc1A	$C_8H_{15}NO_7$	236.0776		
GlcNAc	$C_8H_{15}NO_6$	220.0816	256.0593	
GlcN1A	$C_6H_{13}NO_6$	194.0670		
KDG	$C_6H_{10}O_6$	177.0405		
KDG-6-P	$C_6H_{11}O_9P$	257.0068		263.0144
ADP	$C_{10}H_{15}N_5O_{10}P_2$	426.0221		
AMP	$C_{10}H_{14}N_5O_7P$	346.0558		

Supplementary Table 5 The utilization of different carbon sources by marine *Vibrio* spp. as the sole carbon source.

Strain[a]	Isolation source	The closest relative (16S identity)	Carbon source[b]				Gene/gene cluster[c]	
			β-chitin	Colloidal chitin	GlcNAc	GlcNAc1A	*lpmo*	*ong* cluster
V. sp. D54	Algal sample from the Yellow sea, China	*Vibrio neocaledonicus* NC470 (99.50%)	0.40 ± 0.04	0.58 ± 0.01	0.45 ± 0.02	0.35 ± 0.05	+	+
V. sp. G-C-1	Shrimp from the Arctic	*Vibrio splendidus* ATCC 33125 (98.63%)	0.64 ± 0.06	0.26 ± 0.13	0.36 ± 0.01	0.24 ± 0.01	+	+
V. sp. L5-1	Algal sample from the Yellow sea, China	*Vibrio echinoideorum* NFH.MB010 (98.90%)	0.56 ± 0.10	0.25 ± 0.03	0.43 ± 0.01	0.43 ± 0.03	+	+
V. sp. L3-7	Algal sample from the Arctic	*Vibrio echinoideorum* NFH.MB010 (98.42%)	0.37 ± 0.02	0.49 ± 0.13	0.41 ± 0.01	0.26 ± 0.01	+	+
V. sp. A2-1	Shrimp from the Yellow sea, China	*Vibrio tasmaniensis* LMG 21574 (99.04%)	0.72 ± 0.01	0.55 ± 0.01	0.65 ± 0.04	0.33 ± 0.13	+	+
V. sp. J2-4	Intertidal sediment from the Yellow sea, China	*Vibrio neocaledonicus* NC470 (99.36%)	1.22 ± 0.01	0.75 ± 0.01	0.64 ± 0.02	ND	+	-
V. sp. J1-1	Intertidal sediment from the Yellow sea, China	*Vibrio neocaledonicus* NC470 (99.22%)	ND	0.81 ± 0.01	0.61 ± 0.04	ND	-	-
V. sp. A1-1	Shrimp from the Yellow sea, China	*Vibrio neocaledonicus* NC470 (99.58%)	1.52 ± 0.12	0.80 ± 0.01	0.65 ± 0.03	ND	+	-
V. sp. J2-3	Intertidal sediment from the Yellow sea, China	*Vibrio neocaledonicus* NC470 (99.15%)	1.71 ± 0.02	0.63 ± 0.01	0.63 ± 0.05	ND	+	-
V. sp. CK2-1	Shrimp from the Arctic	*Vibrio rumoiensis* S-1 (99.40%)	0.83 ± 0.01	0.38 ± 0.01	0.30 ± 0.05	ND	+	-
V. sp. A1-b2	Ulvaceae from the Yellow sea, China	*Vibrio plantisponsor* MSSRF60 (99.63%)	ND	0.37 ± 0.03	0.37 ± 0.02	ND	-	-

[a] All *Vibrio* strains examined in this study were isolated by our laboratory.

[b] Bacterial strains with no detectable growth on a given carbon source after 14-day cultivation at 25°C were labeled by “ND”; for bacterial strains grown on a given carbon source, the maximum OD_{600} values of bacterial cells on GlcNAc and GlcNAc1A at 25°C were shown, and the OD_{600} values of their 14-day cultures on β-chitin as well as the OD_{600} values of 4-day cultures on colloidal chitin at 25°C were shown. All data shown are mean ± SD (n = 2 independent experiments). Source data are provided as a Source Data file.

[c] +, presence; -, absence.

Supplementary Table 6 Plasmids used or constructed for genetic manipulations of *P. prydzensis* ACAM 620.

Plasmid	Phenotype
pK18mobsacB-Ery	shuttle vector, kanamycin and erythromycin resistance
pEV	complementary plasmid, ampicillin and chloramphenicol resistance
pK18*lpmo*	knockout vector for LPMO, pK18mobsacB-Ery carrying the upstream and downstream homologous arms of *lpmo*
pK18*ongA*	knockout vector for OngA, pK18mobsacB-Ery carrying the upstream and downstream homologous arms of *ongA*
pK18*ongB*	knockout vector for OngB, pK18mobsacB-Ery carrying the upstream and downstream homologous arms of *ongB*
pK18*ongC*	knockout vector for OngC, pK18mobsacB-Ery carrying the upstream and downstream homologous arms of *ongC*
pK18*ongD*	knockout vector for OngD, pK18mobsacB-Ery carrying the upstream and downstream homologous arms of *ongD*
pK18*kdgK*	knockout vector for KdgK, pK18mobsacB-Ery carrying the upstream and downstream homologous arms of *kdgK*
pK18*ongIT*	knockout vector for OngIT, pK18mobsacB-Ery carrying the upstream and downstream homologous arms of *ongIT*
pK18*ongOT-1*	knockout vector for OngOT-1, pK18mobsacB-Ery carrying the upstream and downstream homologous arms of *ongOT-1*
pK18*ongOT-2*	knockout vector for OngOT-2, pK18mobsacB-Ery carrying the upstream and downstream homologous arms of *ongOT-2*
pK18*ongR*	knockout vector for OngR, pK18mobsacB-Ery carrying the upstream and downstream homologous arms of *ongR*
pEV*ongA*	complementary plasmid for Δ*ongA*, pEV carrying the coding sequence of OngA
pEV*ongB*	complementary plasmid for Δ*ongB*, pEV carrying the coding sequence of OngB
pEV*ongC*	complementary plasmid for Δ*ongC*, pEV carrying the coding sequence of OngC
pEV*ongD*	complementary plasmid for Δ*ongD*, pEV carrying the coding sequence of OngD
pEV*kdgK*	complementary plasmid for Δ*kdgK*, pEV carrying the coding sequence of KdgK
pEV*ongIT*	complementary plasmid for Δ*ongIT*, pEV carrying the coding sequence of OngIT
pEV*ongOT-1*	complementary plasmid for Δ*ongOT-1*, pEV carrying the coding sequence of OngOT-1
pEV*ongOT-2*	complementary plasmid for Δ*ongOT-2*, pEV carrying the coding sequence of OngOT-2
pEV*ongR*	complementary plasmid for Δ*ongR*, pEV carrying the coding sequence of OngR

Supplementary Table 7 Strains used or constructed for genetic manipulations of *P. prydzensis* ACAM 620.

Strain	Phenotype
Escherichia coli WM3064	conjugation donor strain
P. prydzensis ACAM 620	wild-type strain with chitin- and oxidized chitooligosaccharide-degrading abilities
Δ*lpmo*	*lpmo* gene deletion mutant of ACAM 620
Δ*ongA*	*ongA* gene deletion mutant of ACAM 620
Δ*ongA*/pEV*ongA*	the complementary strain of Δ*ongA*
Δ*ongA*/pEV	control strain of Δ*ongA* carrying the empty plasmid pEV
Δ*ongB*	*ongB* gene deletion mutant of ACAM 620
Δ*ongB*/pEV*ongB*	the complementary strain of Δ*ongB*
Δ*ongB*/pEV	control strain of Δ*ongB* carrying the empty plasmid pEV
Δ*ongC*	*ongC* gene deletion mutant of ACAM 620
Δ*ongC*/pEV*ongC*	the complementary strain of Δ*ongC*
Δ*ongC*/pEV	control strain of Δ*ongC* carrying the empty plasmid pEV
Δ*ongD*	*ongD* gene deletion mutant of ACAM 620
Δ*ongD*/pEV*ongD*	the complementary strain of Δ*ongD*
Δ*ongD*/pEV	control strain of Δ*ongD* carrying the empty plasmid pEV
Δ*kdgK*	*kdgK* gene deletion mutant of ACAM 620
Δ*kdgK*/pEV*kdgK*	the complementary strain of Δ*kdgK*
Δ*kdgK*/pEV	control strain of Δ*kdgK* carrying the empty plasmid pEV
Δ*ongIT*	*ongIT* gene deletion mutant of ACAM 620
Δ*ongIT*/pEV*ongIT*	the complementary strain of Δ*ongIT*
Δ*ongIT*/pEV	control strain of Δ*ongIT* carrying the empty plasmid pEV
Δ*ongOT-1*	*ongOT-1* gene deletion mutant of ACAM 620
Δ*ongOT-2*	*ongOT-2* gene deletion mutant of ACAM 620
Δ*ongOT-1*/Δ*ongOT-2*	strain ACAM 620 with genes *ongOT-1* and *ongOT-2* deleted
Δ*ongOT-1*/Δ*ongOT-2*/pEV	control strain of Δ*ongOT-1*/Δ*ongOT-2* carrying the empty plasmid pEV
Δ*ongOT-1*/Δ*ongOT-2*/pEV*ongOT-1*	the complementary strain of Δ*ongOT-1*/Δ*ongOT-2* with pEV*ongOT-1*
Δ*ongOT-1*/Δ*ongOT-2*/pEV*ongOT-2*	the complementary strain of Δ*ongOT-1*/Δ*ongOT-2* with pEV*ongOT-2*
Δ*ongR*	*ongR* gene deletion mutant of ACAM 620
Δ*ongR*/pEV*ongR*	the complementary strain of Δ*ongR*
Δ*ongR*/pEV	control strain of Δ*ongR* carrying the empty plasmid pEV

Supplementary References

1 Vaaje-Kolstad, G. *et al.* An oxidative enzyme boosting the enzymatic conversion of recalcitrant polysaccharides. *Science* **330**, 219-222 (2010).

2 Skane, A. *et al.* The fish pathogen *Aliivibrio salmonicida* LFI1238 can degrade and metabolize chitin despite gene disruption in the chitinolytic pathway. *Appl. Environ. Microbiol.* **87**, e0052921 (2021).

3 Heuts, D. P., Winter, R. T., Damsma, G. E., Janssen, D. B. & Fraaije, M. W. The role of double covalent flavin binding in chito-oligosaccharide oxidase from *Fusarium graminearum*. *J. Biol. Chem.* **413**, 175-183 (2008).

4 Cummings, J. A. *et al.* Annotating enzymes of uncertain function: the deacylation of D-amino acids by members of the amidohydrolase superfamily. *Biochemistry* **48**, 6469-6481 (2009).

5 Lai, W. L. *et al.* The functional role of the binuclear metal center in D-aminoacylase: one-metal activation and second-metal attenuation. *J. Biol. Chem.* **279**, 13962-13967 (2004).
