# Supplementary material for: A pathway for chitin oxidation in marine bacteria
Source: Nat Commun. 2022 Oct 6;13:5899. doi: 10.1038/s41467-022-33566-5 (PMC9537276; doi:10.1038/s41467-022-33566-5)
Supplement: Supplementary file 2 — Description of Additional Supplementary Files [file 41467_2022_33566_MOESM2_ESM.docx]

**Description of Additional Supplementary Files**

File Name: Supplementary Data 1

Description: A list of all proteins identified in the secretome of strain ACAM 620 grown on colloidal chitin as the sole carbon source.

File Name: Supplementary Data 2

Description: Distribution of genes involved in the oxidative chitin utilization pathway in marine bacterial strains.

File Name: Supplementary Data 3

Description: Distribution of genes involved in the oxidative chitin utilization pathway in terrestrial bacterial strains.

File Name: Supplementary Data 4

Description: Distribution of genes involved in the oxidative chitin utilization pathway in human-associated bacteria.

File Name: Supplementary Data 5

Description: Primers used in this study.
