# Supplementary material for: A pathway for chitin oxidation in marine bacteria
Source: Nat Commun. 2022 Oct 6;13:5899. doi: 10.1038/s41467-022-33566-5 (PMC9537276; doi:10.1038/s41467-022-33566-5)
Supplement: Supplementary file 8 — Reporting Summary [file 41467_2022_33566_MOESM8_ESM.pdf]

nature portfolio

Corresponding author(s): Ping-Yi Li; Yu-Zhong Zhang

Last updated by author(s): Sept 21, 2022

# Reporting Summary

Nature Portfolio wishes to improve the reproducibility of the work that we publish. This form provides structure for consistency and transparency in reporting. For further information on Nature Portfolio policies, see our Editorial Policies and the Editorial Policy Checklist.

## Statistics

For all statistical analyses, confirm that the following items are present in the figure legend, table legend, main text, or Methods section.

| n/a | Confirmed | |
|---|---|---|
| ☐ | ☒ | The exact sample size ($n$) for each experimental group/condition, given as a discrete number and unit of measurement |
| ☐ | ☒ | A statement on whether measurements were taken from distinct samples or whether the same sample was measured repeatedly |
| ☒ | ☐ | The statistical test(s) used AND whether they are one- or two-sided<br>*Only common tests should be described solely by name; describe more complex techniques in the Methods section.* |
| ☒ | ☐ | A description of all covariates tested |
| ☒ | ☐ | A description of any assumptions or corrections, such as tests of normality and adjustment for multiple comparisons |
| ☐ | ☒ | A full description of the statistical parameters including central tendency (e.g. means) or other basic estimates (e.g. regression coefficient) AND variation (e.g. standard deviation) or associated estimates of uncertainty (e.g. confidence intervals) |
| ☒ | ☐ | For null hypothesis testing, the test statistic (e.g. $F$, $t$, $r$) with confidence intervals, effect sizes, degrees of freedom and $P$ value noted<br>*Give P values as exact values whenever suitable.* |
| ☒ | ☐ | For Bayesian analysis, information on the choice of priors and Markov chain Monte Carlo settings |
| ☒ | ☐ | For hierarchical and complex designs, identification of the appropriate level for tests and full reporting of outcomes |
| ☒ | ☐ | Estimates of effect sizes (e.g. Cohen's $d$, Pearson's $r$), indicating how they were calculated |

*Our web collection on statistics for biologists contains articles on many of the points above.*

## Software and code

Policy information about availability of computer code

| | |
|---|---|
| Data collection | Genomes were sequenced using the Illumina HiSeq2000 platform and genomic data were processed using Trimmomatic version 0.36, SPAdes version 3.11.1 and GapCloser version 1.1.<br>Transcriptomes were sequenced using the Illumina HiSeq2000 platform and transcriptional data were processed using Rockhopper version 2.0.3.<br>RT-qPCR was performed using the LightCycler®480 software version 1.5.1.62.<br>The MS data of enzymatic reaction products were collected using OtofControl version 3.4. |
| Data analysis | Bacterial genomes were annotated by the on-line RAST v2.0 server.<br>Transcriptional data were analyzed using Rockhopper version 2.0.3.<br>Identification and quantification of secreted proteins were conducted by MaxQuant v1.5.<br>The RT-qPCR data were analyzed using the LightCycler®480 software version 1.5.1.62 with the “Advanced Relative Quantification” method.<br>The MS data of enzymatic reaction products were analyzed using DataAnalysis 4.2.<br>Cellular location of proteins was predicted by PSORTb v3.0 and CELLO v.2.5 combined with SignalP 5.0.<br>Phylogenetic analyses of proteins were aligned by MUSCLE version 3.8.31 and visualized using Molecular Evolutionary Genetics Analysis version 7.0.<br>Software for initial data processing was Microsoft Excel 2016, and subsequent analyses were carried out using OriginPro 8.5 v.b161 and OriginPro 2022b v.9.9.5.171. |

For manuscripts utilizing custom algorithms or software that are central to the research but not yet described in published literature, software must be made available to editors and reviewers. We strongly encourage code deposition in a community repository (e.g. GitHub). See the Nature Portfolio guidelines for submitting code & software for further information.

## Data

Policy information about availability of data

All manuscripts must include a data availability statement. This statement should provide the following information, where applicable:

- Accession codes, unique identifiers, or web links for publicly available datasets
- A description of any restrictions on data availability
- For clinical datasets or third party data, please ensure that the statement adheres to our policy

All data supporting the findings of this study are available within the paper (and its Supplementary Information files). Genome sequences obtained in this study have been deposited in GenBank/DDBJ under accession numbers CP091442 (https://www.ncbi.nlm.nih.gov/assembly/GCF_021729425.1/), CP091443 (https://www.ncbi.nlm.nih.gov/assembly/GCF_021729425.1/), JAKKCI000000000 (https://www.ncbi.nlm.nih.gov/assembly/GCF_021726015.1/), JAKKCJ000000000 (https://www.ncbi.nlm.nih.gov/assembly/GCF_021726515.1/), JAKKCK000000000 (https://www.ncbi.nlm.nih.gov/assembly/GCF_021725735.1/), JAKKCL000000000 (https://www.ncbi.nlm.nih.gov/assembly/GCF_021728175.1/), JAKKCM000000000 (https://www.ncbi.nlm.nih.gov/assembly/GCF_021728395.1/), JAKKCN000000000 (https://www.ncbi.nlm.nih.gov/assembly/GCF_021728155.1/), JAKKCO000000000 (https://www.ncbi.nlm.nih.gov/assembly/GCF_021728355.1/), JAKKCP000000000 (https://www.ncbi.nlm.nih.gov/assembly/GCF_021727995.1/), JAKKCQ000000000 (https://www.ncbi.nlm.nih.gov/assembly/GCF_021728315.1/), JAKKCR000000000 (https://www.ncbi.nlm.nih.gov/assembly/GCF_021728015.1/), JAKKCS000000000 (https://www.ncbi.nlm.nih.gov/assembly/GCF_021728075.1/), JAKKCT000000000 (https://www.ncbi.nlm.nih.gov/assembly/GCF_021728095.1/), JAKKCU000000000 (https://www.ncbi.nlm.nih.gov/assembly/GCF_021728055.1/), JAKKCV000000000 (https://www.ncbi.nlm.nih.gov/assembly/GCF_021728035.1/), and JAKKCW000000000 (https://www.ncbi.nlm.nih.gov/assembly/GCF_021728455.1/). All the RNA-seq read data have been deposited in NCBI's sequence read archive (SRA) under project accession number PRJNA196223 (https://www.ncbi.nlm.nih.gov/bioproject/PRJNA196223/). The mass spectrometry proteomics data have been deposited in the ProteomeXchange Consortium via the PRIDE with identifier PXD031176 (https://www.ebi.ac.uk/pride/archive/projects/PXD031176). A reporting summary for this article is available as a Supplementary Information file. Source data are provided with this paper.

## Human research participants

Policy information about studies involving human research participants and Sex and Gender in Research.

| | |
|---|---|
| Reporting on sex and gender | N/A |
| Population characteristics | N/A |
| Recruitment | N/A |
| Ethics oversight | N/A |

Note that full information on the approval of the study protocol must also be provided in the manuscript.

# Field-specific reporting

Please select the one below that is the best fit for your research. If you are not sure, read the appropriate sections before making your selection.

☒ Life sciences ☐ Behavioural & social sciences ☐ Ecological, evolutionary & environmental sciences

For a reference copy of the document with all sections, see nature.com/documents/nr-reporting-summary-flat.pdf

# Life sciences study design

All studies must disclose on these points even when the disclosure is negative.

| | |
|---|---|
| Sample size | No sample size calculations were performed for this study. Sample sizes are indicated for each experiment and were chosen based on similar studies. |
| Data exclusions | No data was excluded from the analysis. |
| Replication | All experiments were repeated 3 times as completely independent experiments unless otherwise is stated. All attempts at replication were successful. |
| Randomization | The designed experiments in this study didn't include random experiments, and the experimental results were not randomized. |
| Blinding | Blinding is not relevant to this study: only one variable is tested in each experiment. |

# Reporting for specific materials, systems and methods

We require information from authors about some types of materials, experimental systems and methods used in many studies. Here, indicate whether each material, system or method listed is relevant to your study. If you are not sure if a list item applies to your research, read the appropriate section before selecting a response.

## Materials & experimental systems

| n/a | Involved in the study |
|---|---|
| ☒ | ☐ Antibodies |
| ☒ | ☐ Eukaryotic cell lines |
| ☒ | ☐ Palaeontology and archaeology |
| ☒ | ☐ Animals and other organisms |
| ☒ | ☐ Clinical data |
| ☒ | ☐ Dual use research of concern |

## Methods

| n/a | Involved in the study |
|---|---|
| ☒ | ☐ ChIP-seq |
| ☒ | ☐ Flow cytometry |
| ☒ | ☐ MRI-based neuroimaging |
